# A genetically informed longitudinal study of early-life temperament and childhood aggression

**DOI:** 10.1017/S0954579424000634

**Published:** 2025-05

**Authors:** Eric N. Penichet, Christopher R. Beam, Susan E. Luczak, Deborah W. Davis

**Affiliations:** 1 Department of Psychology, University of Southern California, Los Angeles, CA, USA; 2 School of Geronotology, University of Southern California, Los Angeles, CA, USA; 3 Department of Pediatrics, University of Louisville School of Medicine, Louisville, KY, USA; 4 Norton Children’s Research Institute affiliated with the University of Louisville School of Medicine, Louisville, KY, USA

**Keywords:** aggression, behavior genetics, childhood, externalizing behavior, temperament

## Abstract

The present study examined the longitudinal associations between three dimensions of temperament – activity, affect-extraversion, and task orientation – and childhood aggression. Using 131 monozygotic and 173 dizygotic (86 same-sex) twin pairs from the Louisville Twin Study, we elucidated the ages, from 6 to 36 months, at which each temperament dimension began to correlate with aggression at age 7. We employed latent growth modeling to show that developmental increases (i.e., slopes) in activity were positively associated with aggression, whereas increases in affect-extraversion and task orientation were negatively associated with aggression. Genetically informed models revealed that correlations between temperament and aggression were primarily explained by common genetic variance, with nonshared environmental variance accounting for a small proportion of each correlation by 36 months. Genetic variance explained the correlations of the slopes of activity and task orientation with aggression. Nonshared environmental variance accounted for almost half of the correlation between the slopes of affect-extraversion and aggression. Exploratory analyses revealed quantitative sex differences in each temperament-aggression association. By establishing which dimensions of temperament correlate with aggression, as well as when and how they do so, our work informs the development of future child and family interventions for children at highest risk of aggression.

Childhood aggression, often regarded as an indicator of maladjustment (Card et al., 2008), is a multidetermined developmental outcome. Typically, physical aggression emerges during infancy, peaks between the ages of 2 and 4 years, and then declines as the capacities for reasoning and self-regulation develop (Côté et al., 2006; Hay et al., 2014; Tremblay et al., 1999, 2018). Yet for some children, aggressive behavior can lead to peer rejection (Little & Garber, 1995; Parker & Asher, 1987), academic difficulties (Bierman et al., 2013; Farmer & Bierman, 2002; Turney & McLanahan, 2015), strained family relationships (Morelli et al., 2022; Patterson, 1982), and delinquent behavior (Hay et al., 2017; Roff, 1992; Roff & Wirt, 1984). Moreover, childhood aggression is also associated with a wide range of adverse mental health outcomes, including depression (Capaldi, 1991; Weiss & Catron, 1994) and anxiety (Crick et al., 2006; Granic, 2014). Childhood aggression is also a foundational component of conduct disorder (American Psychiatric Association, 2013; Nock et al., 2006), presenting a substantial disease burden among children worldwide (Erskine et al., 2014). Early detection of aggressive tendencies is a priority among educators, psychologists, and parents, as it provides the best possible chance to intervene and promote healthier child development.

To elucidate early correlates of childhood aggression, the present study examines whether, when, and how different dimensions of infant and toddler temperament are associated with aggression at age 7. Using observer measures from the Infant Behavior Record (Bayley, 1969), we evaluate the temperament dimensions of activity, affect-extraversion, and task orientation, collectively encompassing reactive and self-regulatory facets of temperament, as possible correlates of aggression. In addition to determining the ages, from 6 to 36 months, at which correlations emerge, we perform latent growth curve analyses to test whether variance in developmental increases (i.e., mean-level change) in each temperament dimension explains individual differences in childhood aggression. We leverage our genetically informative sample to interrogate the genetic and environmental variance underlying the associations between temperament and aggression. Finally, we evaluate quantitative sex differences in the associations between each temperament dimension and aggression, that is, whether boys and girls differ in the magnitudes of the genetic and environmental influences underlying these associations.

## Dimensions of temperament and childhood aggression

Temperament refers to early-emerging, biologically based differences in behavioral tendencies (Goldsmith et al., 1987). Temperament theorists posit that the expression of temperament is at its purest during infancy and that after this period, isolating the expression of temperament versus that of other behaviors is more challenging (Goldsmith et al., 1987). As such, infancy and toddlerhood represent a critical period through which to examine temperament and its longitudinal associations with aggression. Presently, we focus on three facets of temperament that have theoretical and empirical associations with childhood aggression: effortful control, activity, and sociability.

Effortful control, which refers to a child’s capacity to regulate impulses and behaviors (Rothbart & Bates, 2006), has been found to be negatively associated with childhood aggression (Rothbart et al., 1994) and broader externalizing behavior issues (Valiente et al., 2003; Wilson et al., 2021). Specifically, cross-sectional studies have reported significant negative associations between effortful control, including related traits (e.g., inhibitory control), and externalizing behavior in early childhood (Nwadinobi & Gagne, 2020; Olson et al., 2005; Scheper et al., 2017) and middle childhood (Eisenberg et al., 2001). Moreover, longitudinal studies have found that effortful control in infancy and toddlerhood negatively correlates with externalizing behavior, including aggression, in early childhood (Gartstein et al., 2012; Kochanska & Knaack, 2003; Murray & Kochanska, 2002). In particular, one study identified that 6 month-old infants’ “looking away response,” which indexes effortful control during situations where the child needs to regulate frustration, was negatively associated with aggression two years later (Crockenberg et al., 2008). Another study found that middle childhood impulsive behavior, which is implicated in low effortful control, was positively associated with externalizing problems in adolescence (Olson et al., 1999).

Activity, referring to the vigor in a child’s bodily motor movement and expenditure of energy (Buss & Plomin, 1984), is positively associated with childhood aggression (Nwadinobi & Gagne, 2020; Schaughency & Fagot, 1993) and broader externalizing behavior issues (Fagot & O’Brien, 1994; Gartstein et al., 2012; Scheper et al., 2017; Teglasi & MacMahon, 1990). Specifically, cross-sectional studies have found significant positive associations between activity and externalizing problems, including aggression, in toddlerhood (Fagot & O’Brien, 1994), early childhood (De Pauw et al., 2009; Gartstein et al., 2012; Nwadinobi & Gagne, 2020; Schaughency & Fagot, 1993; Scheper et al., 2017), and middle childhood (Gjone & Stevenson, 1997; Teglasi & MacMahon, 1990). Longitudinal studies have further found that activity in the first year of life is positively associated with externalizing problems in early to late childhood (Lahey et al., 2008; Morales et al., 2022). Moreover, high levels of activity in infancy have also been positively associated with childhood impulsivity and inattention (Olson et al., 2002), both of which are correlates of aggressive behavior (Olson et al., 1999; Yoo et al., 2021).

Sociability, conceptualized as a facet of the broader temperament dimension of surgency/extraversion (Putnam et al., 2006), refers to a child’s propensity to seek and prefer social interaction rather than being alone (Cheek & Buss, 1981). Cross-sectional studies during early and middle childhood have generally found small positive associations between sociability and externalizing problems, including aggression (Gjone & Stevenson, 1997; Russell et al., 2003). In contrast, longitudinal studies suggest negative associations between early-life sociability and externalizing problems, showing that socially withdrawn infants, toddlers, and preschoolers exhibit higher levels of externalizing behavior, including aggression, in childhood (Chen et al., 2015; Guedeney et al., 2014; Liang et al., 2019). Interestingly, in the longitudinal literature, it has also been reported that surgency/extraversion, of which sociability is a component, is positively associated with externalizing behavior (Berdan et al., 2008; Gunnar et al., 2003; Rothbart et al., 2001). This apparent inconsistency between longitudinal studies suggesting that sociability is a protective factor for behavior problems versus studies suggesting that surgency/extraversion is a risk factor may be attributed to the inclusion of known correlates of externalizing behavior, such as high-activity level (Lahey et al., 2008) and high-intensity pleasure-seeking (Oldehinkel et al., 2004), in surgency/extraversion.

## Theoretical model of temperament and childhood aggression

The spectrum model is an empirically supported model that has been used to understand how features of children’s temperament and behavior overlap with psychopathology, including aggressive behavior. The spectrum model posits that temperament and psychopathology are situated on a dimensional continuum, with temperament a subclinical manifestation of the latter, linked by common etiological influences (Lemery-Chalfant & Clifford, 2020; Tackett, 2006). The spectrum model has traditionally received support from twin studies (Gagne et al., 2011; Lemery-Chalfant et al., 2008) and psychobiological studies (Beauchaine et al., 2001; Iacono et al., 1999), which, respectively, have found genetic variance and psychobiological correlates shared across self-regulatory traits and externalizing psychopathology. Common genetic variance, however, need not be the only explanation for the association between temperament and aggression, suggesting that longitudinal studies of temperament are needed to test the spectrum model (Lemery-Chalfant & Clifford, 2020). For instance, nurturing childhood environmental exposures, such as warm and responsive parenting, exhibit positive associations with effortful control (Eiden et al., 2004) and negative associations with aggression (Bayer & Cegala, 1992; Boeldt et al., 2012). Thus, given that the home environment may influence both temperament and aggression, a longitudinal study examining the extent to which genetic versus environmental variance underlies the association between early-life temperament and childhood aggression may provide a more comprehensive assessment of the spectrum model.

## Behavior genetic literature on temperament and aggression

As noted above, behavior genetic (“twin”) studies have been used to explicate the genetic and environmental sources of variance underlying temperament and aggression. These studies provide the clearest evidence that genetic variance accounts for the association between dimensions of infant and toddler temperament and childhood aggression. Much of this research, however, has focused on the temperament dimension of negative emotionality, which refers to a child’s tendency to experience negative emotions such as sadness, anger, fear, and irritability (Rothbart & Bates, 2006). These studies have found that common genetic factors account for the association between early childhood negative emotionality and externalizing behavior, including aggression (Mikolajewski et al., 2013; Schmitz et al., 1999; Singh & Waldman, 2010), with some evidence of unique genetic influences (Mikolajewski et al., 2013; Singh & Waldman, 2010). Fewer studies, however, have evaluated the genetic and environmental associations underlying other temperament dimensions and aggression. As we next review, although effortful control has been studied, both activity and sociability, despite their associations with aggression, remain understudied using twin and sibling studies.

Effortful control and related constructs, such as attentional regulation and inhibitory control, have been found to exhibit moderate to substantial genetic overlap with externalizing behavior problems, including aggression, in toddlerhood (Gagne et al., 2011, 2020), early and middle childhood (Deater-Deckard et al., 2007; Lemery-Chalfant et al., 2008), and adolescence (Vasin & Lobaskova, 2016). Despite observed genetic correlations between effortful control and behavior problems, unique genetic variance has been found to underlie both traits. Moreover, one study found evidence of common nonshared environmental variance underlying the association between inhibitory control, a subordinate dimension of effortful control, and externalizing behavior (Gagne et al., 2011). Another study found that common genetic and shared environmental variance accounted for the association between toddlerhood self-control and externalizing problems (Rhee et al., 2018). It should be noted, however, that the self-control measure used in this study, consisting of a “do not touch” compliance task measuring the number of seconds a child could refrain from touching an attractive toy (Rhee et al., 2018), is more substantively and contextually specific than temperamental effortful control, and, as such, direct comparisons between both constructs must be qualified.

Although the association between activity and childhood aggression is well established (Fagot & O’Brien, 1994; Scheper et al., 2017; Teglasi & MacMahon, 1990), the genetic and environmental factors that underlie this association remain understudied. The only twin study to explore the common etiology of the association between activity and childhood aggression did not find common genetic or shared environmental variance accounting for their covariance (Gjone & Stevenson, 1997). A more recent study that focused on symptoms of attention-deficit/hyperactivity disorder (ADHD) showed a moderate genetic correlation between motor activity and parent-rated attention problems in toddlerhood (Saudino et al., 2018). Relatedly, another study found that common genetic variance accounted for the association between infants’ activity level and ADHD symptoms (Ilott et al., 2010). Taken together, common genetic variance may account for the association between activity and aggression.

Despite literature suggesting negative associations between infant and toddler sociability and childhood externalizing problems (Chen et al., 2015; Guedeney et al., 2014; Liang et al., 2019), no study has investigated the genetic and environmental factors underlying these associations. One study focusing on the etiology of the association between approach/positive anticipation and childhood aggression showed that both genetic and nonshared environmental factors contributed to this association (Deater-Deckard et al., 2010). Given these findings, and the substantial heritability of sociability (Goldsmith et al., 1997; Saudino et al., 1995) and the moderate to substantial heritability of aggression (Porsch et al., 2016; Tuvblad & Baker, 2011), common genetic influences may underlie the association between sociability and aggression.

Up to this point, we have only reported whether and to what extent common genetic and environmental variance accounts for the associations between dimensions of temperament and childhood aggression. Most twin studies focus on one or two time points, or aggregate data across multiple time points, to investigate the etiological components of the association between temperament and aggression (Deater-Deckard et al., 2007; Gagne et al., 2011, 2020; Lemery-Chalfant et al., 2008; Singh & Waldman, 2010). An unaddressed question in this literature concerns how common genetic and environmental variance accounts for the temporal association between dimensions of temperament and childhood aggression. Existing research has shown that temperament and externalizing behavior problems show greater effects of common genetic variance in cross-sectional versus longitudinal studies (Deater-Deckard et al., 2007; Gagne et al., 2011, 2020), implying that when temperament and externalizing behavior are measured closely together in time, concurrently expressed genetic influences may contribute to their stronger genetic correlations. Thus, in the present study, we use six measurements of temperament – at 6, 12, 18, 24, 30, and 36 months – and one measurement of aggression at age 7 to test a spectrum conceptualization of the associations between three temperament dimensions and aggression, in which common genetic variance accounts for these associations. Given that the transition from late toddlerhood to early childhood is often characterized by an increased exposure to a greater diversity of environments and experiences, such as the start of preschool and expanded social interactions (Malik & Marwaha, 2022), we were especially interested in exploring whether nonshared environmental variance would begin to explain part of the association between temperament and aggression.

## Developmental trajectories of temperament and childhood aggression

Although prior research has shown that both genetic and environmental sources of variance account for the bivariate association between dimensions of temperament and aggressive behavior, no study has examined the shared etiology underlying the developmental trajectories of temperament and childhood aggression. The importance of employing a longitudinal method becomes evident if we consider, for example, the temperament dimension of effortful control, which consists of higher-order cognitive domains that begin to emerge towards the end of the first year of life when frontal regions underlying executive function start to develop (Posner & Raichle, 1994; Posner & Rothbart, 2007; Rothbart & Jones, 1998). Given the dynamic nature of this temperament dimension and its progressive growth during the early years of life (Kochanska et al., 2000; Li-Grining, 2007), developmental increases in effortful control may serve as a protective factor against childhood aggression. Consistent with this notion, increases in toddlerhood self-control (a construct related to effortful control) from 14 to 36 months, but not initial levels, have been found to be associated with fewer externalizing problems, with both genetic and shared environmental factors influencing this association (Rhee et al., 2018). In contrast, null associations between infant sociability trajectories and behavior problems have been found elsewhere (Kim, 2022). Notably, none of these studies examined childhood aggression as an outcome, nor considered the developmental change in the temperament dimension of activity as a possible correlate. Thus, a secondary aim of the current study is to test (a) whether differences in intraindividual change in dimensions of temperament are associated with childhood aggression and (b) whether genetic variance, environmental variance, or both account for the association between change in temperament and aggression.

## Sex differences in the association between dimensions of temperament and aggression

Quantitative sex differences, or differences in the degree to which a trait is expressed between boys and girls, have been found for both temperament and aggression. Specifically, several studies have reported quantitative sex differences for activity (Campbell & Eaton, 1999; Eaton & Yu, 1989; Goggin, 1975), effortful control (Else-Quest et al., 2006; Gagne et al., 2013), and sociability (Gunnar & Donahue, 1980; Olino et al., 2013), showing that boys exhibit higher levels of activity, lower levels of effortful control, and lower levels of sociability than girls. Boys have also been shown to exhibit higher levels of aggression than girls (Eme, 2016; Mayes et al., 2020). Importantly, several twin studies have also explored sex differences in the magnitudes of genetic and environmental influences underlying aggression, with some studies reporting greater genetic variance among boys (Hudziak et al., 2003; van Beijsterveldt et al., 2003) and greater shared and nonshared environmental variance among girls (Baker et al., 2008), whereas others have found no such distinctions (Eley et al., 1999; Tuvblad et al., 2009). Fewer twin studies, however, have explored quantitative sex differences underlying temperament, with the study closest in scope reporting differences in heritability estimates across boys and girls for the Alienation, Control, and Absorption scales of the Multiple Dimensions Personality Questionnaire (Finkel & McGue, 1997), with boys exhibiting higher heritability estimates for Alienation and Control and lower heritability estimates for Absorption (Finkel & McGue, 1997). Given the lack of twin studies exploring quantitative sex differences in temperament, as well as the association between temperament and aggression, the present study also tests for sex differences in the magnitudes of genetic and environmental influences underlying associations between temperament and aggression.

## Present study

Using longitudinal measures of temperament and childhood aggression data from the Louisville Twin Study, we examined the genetic and environmental variance underlying the phenotypic associations between three temperament dimensions – activity, affect-extraversion, and task orientation – from 6 to 36 months of age and childhood aggression at 84 months (7 years). As we discuss in greater detail in the Methods section, the temperament dimensions of affect-extraversion and task orientation, ascertained using the Infant Behavior Record, bear conceptual resemblance to the more broadly recognized dimensions of sociability and effortful control, respectively.

We first hypothesized that the longitudinal associations between each temperament dimension and aggression at age 7 would be increasingly correlated from 6 months to 36 months. Based on the temperament literature, we predicted that activity would be positively associated with aggression, whereas affect-extraversion and task orientation would be negatively associated with aggression. Second, we hypothesized that individual differences in mean-level increases in each temperament dimension would show significant correlations in the aforementioned directions. Third, in keeping with the spectrum model, we hypothesized that genetic sources of variance would account for the correlations between each temperament dimension and aggression, with nonshared environmental variance beginning to account for part of these associations by 30 months. Based on literature showing greater genetic correlations when temperament and aggression are measured closer in time (Deater-Deckard et al., 2007; Gagne et al., 2011, 2020), as part of this hypothesis, we tested the earliest age at which genetic correlations between each temperament dimension and aggression was statistically significant. Finally, given evidence of greater heritability estimates for boys than girls in both aggression (Hudziak et al., 2003; van Beijsterveldt et al., 2003) and self-regulatory dimensions of personality (Finkel & McGue, 1997), as an exploratory hypothesis, we predicted that genetic influences would account for a larger proportion of the covariation between each temperament dimension and aggression in boys, with this effect being particularly salient for temperamental task orientation.

## Methods

### Participants

Participants in the Louisville Twin Study were recruited from birth certificate records provided by the Board of Health, which represented families residing in the Jefferson County, Kentucky region between 1957 and 2000. Families spanned the full socioeconomic spectrum of the Louisville metropolitan region at the time of their recruitment. Of the total sample, 80% were of European ancestry, 18% were African American, and 2% were of mixed or Asian ancestry (Davis et al., 2015). Socioeconomic status (SES) of the household heads, as measured by Duncan’s scores for SES (Hollingshead, 1975), had a mean score of 46.89 (*SD* = 26.9), which was similar to that of middle-level clerical employees (Davis et al., 2015). Mothers and fathers had an average of 13.20 and 13.61 years of education, respectively. The mean gestational age of the sample was 37.4 weeks, which is younger than the general population mean of 40 weeks for neonates but not unusual among twins. In accordance with the LTS protocol, zygosity was determined by analyzing twins’ blood serum when they were 36 months or older.

The present study consisted of 608 individuals (321 girls and 287 boys), including 131 monozygotic (MZ) twin pairs and 173 dizygotic (DZ) twin pairs (86 same-sex, 87 opposite-sex), born from 1977 to 1996. Individuals were included in the study if they had temperament data (collected from age 6 to 36 months) and/or aggression data (collected at 84 months [7 years]). Table 1 displays the total number of individuals with data at each time point. Table A1 in the Supplementary Materials indicates how many twins at each time point from 6 to 36 months also participated at age 7. We also present attrition analyses, further elaborated upon in the Results section, to test whether the subgroup of participants who participated at age 7 was representative of those who participated earlier in terms of gender (i.e., % boys), socioeconomic status, and temperament (Table A2). We present sample sizes for genetically informed models (described later in the methods section) in Table A3 of the Supplementary Materials.

### Measures

#### Childhood aggression

Aggression at age 7 was measured using the aggression subscale of the School Behavior Checklist (SBC; Miller, 1977). The SBC is a 104-item true/false survey of classroom behaviors completed by teachers to convey their impression of children in their classrooms. The items covered a wide range of social and emotional traits, ranging from social competence to moderate social deviance. The aggression subscale, which consisted of 36 items, was made up of both “active” and “passive” aggression items. Examples of active items included “Hits and pushes other children,” “Starts fighting over nothing,” and “Uses abusive language toward other children.” Examples of passive items included “Refusing to speak when angry,” “Sulking when mad,” and “Being stubborn.” The Cronbach’s alpha of the aggression subscale in the current sample was 0.89. We used the sum scores of the aggression subscale, which were the sum of items endorsed true for each child.

#### Infant and toddler temperament

Temperament was measured using Bayley’s Infant Behavior Record (IBR; Bayley, 1969), which was a summary evaluation of a range of infant behaviors observed during the administration of the Mental and Motor Scales of the Bayley Scales of Infant Development (BSID). Given the focus of the present study on infancy and toddlerhood, participants included those who received BSID at 6, 12, 18, 24, 30, and 36 months of age. The IBR was completed in accordance with the BSID handbook (Bayley, 1969) after the administration of the Mental and Motor scales. Based on principal components analysis, five factors were derived from the IBR: activity, affect-extraversion, task orientation, auditory-visual awareness, and motor coordination (Matheny, 1980). The present study included activity, affect-extraversion, and task orientation dimensions. Activity assessed children’s general level of energy and motor activity, using items evaluating gross bodily movement, body motion, and level of energy. Affect-extraversion measured children’s social engagement and emotionality, using items assessing responsiveness to persons, responsiveness to examiner, cooperation with examiner, fearfulness, happiness, object orientation, and behavior constancy. Affect-extraversion is similar to temperamental sociability, both reflecting children’s tendency for positive social engagement. Finally, task orientation, using items evaluating object orientation, goal-directedness, and attention span, measured children’s level of focus and persistence during the administration of the BSID. Given that it reflects children’s degree of attentional and behavioral regulation, task orientation is similar to temperamental effortful control. The Cronbach’s alpha for each of the activity, affect-extraversion, and task orientation subscales in the current sample was 0.86, 0.81, and 0.84, respectively.

We estimated factor scores for each temperament at ages 6, 12, 18, 24, 30, and 36 months instead of summary scores to better capture developmental changes in unbiased measures of temperament over time. Importantly, due to insufficient item-level data for activity at 36 months, factor scores for this temperament dimension were only available up to 30 months. We employed moderated nonlinear factor analysis (MNFLA) to generate age and sex-adjusted factor scores, allowing us to adjust scores for uniform and nonuniform differential item functioning due to sex, age, and their interaction (Curran et al., 2014). We present the results from the final MNFLA model for each temperament dimension in Table A4 of the Supplementary Materials.

### Data analysis

We first computed the phenotypic correlations between temperament dimensions at 6, 12, 18, 24, 30, and 36 months and aggression at age 7. Sex, birth year, and socioeconomic status were included as covariates in these analyses, as well as in subsequent analyses. Attrition analyses revealed no significant differences in gender (i.e., % boys) among children who participated at age 7 versus those who dropped out (Table A2). There were also no significant mean differences in socioeconomic status between both groups. Those who dropped out at age 7, however, showed significantly lower affect-extraversion at age 30 months and task orientation at ages 12 and 24 months than those who participated. Given that significant mean differences did not appear systematic, occurring in 3 out of 17 *t*-tests, these findings suggested that data probably at least met the missing at random assumption. We, therefore, handled missing data using full-information maximum likelihood in M*plus* 8.8 (Enders, 2001; Muthén & Muthén, 1998–2017).

Next, we fitted latent growth models to temperament data from 6 to 36 months to examine interindividual differences in mean-level growth trajectories of activity, affect-extraversion, and task orientation. We evaluated three models: a linear model, a quadratic model, and the latent basis model. In contrast to linear and quadratic models, which tested parametric growth trajectories (i.e., linear and quadratic) for temperament across development, the latent basis model (McArdle & Epstein, 1987; Meredith & Tisak, 1990) treated the growth trajectory of temperament as a latent variable. By allowing factor loadings for the latent slope variable to be freely estimated, the latent basis model utilized the observed temperament data to estimate the optimal growth trajectory, allowing nonlinear changes to be modeled efficiently. Here, the slope consisted of a weighted sum of change defined by the model estimated basis weights and a variance that characterized individual differences in the ‘amplitude’ of change (Ram & Grimm, 2007). By remaining atheoretical about the form of growth, the latent basis model is useful for modeling traits, such as temperament, for which expected growth trajectories have yet to be elucidated. Intercept and slope variances from the best fitting growth model were used in subsequent models, as described below.

Because the latent basis model is not nested within the linear or quadratic growth models, model selection primarily used the Akaike Information Criterion (AIC; Akaike, 1973) and Bayesian Information Criterion (BIC; Schwarz, 1978). AIC and BIC both reward goodness of fit but differ in the degree of penalty for model complexity (Burnham & Anderson, 2004). The AIC penalizes model fit by a constant factor of 2 (i.e., AIC = 2*k* – 2ln[*L*]), whereas the BIC penalizes model fit by the natural log of the number of observations (i.e., BIC = *k*ln[*n*] - 2ln[*L*]). Lower AIC and BIC values indicate better fit. In addition, we examined the Tucker-Lewis Index (TLI; Tucker & Lewis, 1973) and the root mean square error of approximation (RMSEA; Browne & Cudeck, 1992), particularly when comparing the linear and quadratic growth models. TLI compares the specified model with a null model, with values closer to 1 indicating better fit. RMSEA estimates the lack of fit per degree of freedom, with values closer to 0 indicating better fit.

Next, twin correlations were calculated for (1) each temperament dimension (activity, affect-extraversion, task orientation) at each age of measurement; (2) their respective slope and intercept factors; and (3) aggression at age 7. Larger MZ correlations than DZ correlations indicate that genetic variance underlies each temperament dimension and aggression. To the extent that the MZ correlations are less than unity, nonshared environmental variance underlies each trait. DZ correlations greater than half of the MZ correlations suggest shared environmental variance underlying each trait. We also calculated the cross-trait cross-twin correlations to infer the shared etiology of temperament and aggression, which are interpreted identically as univariate twin correlations.

**Figure 1.**
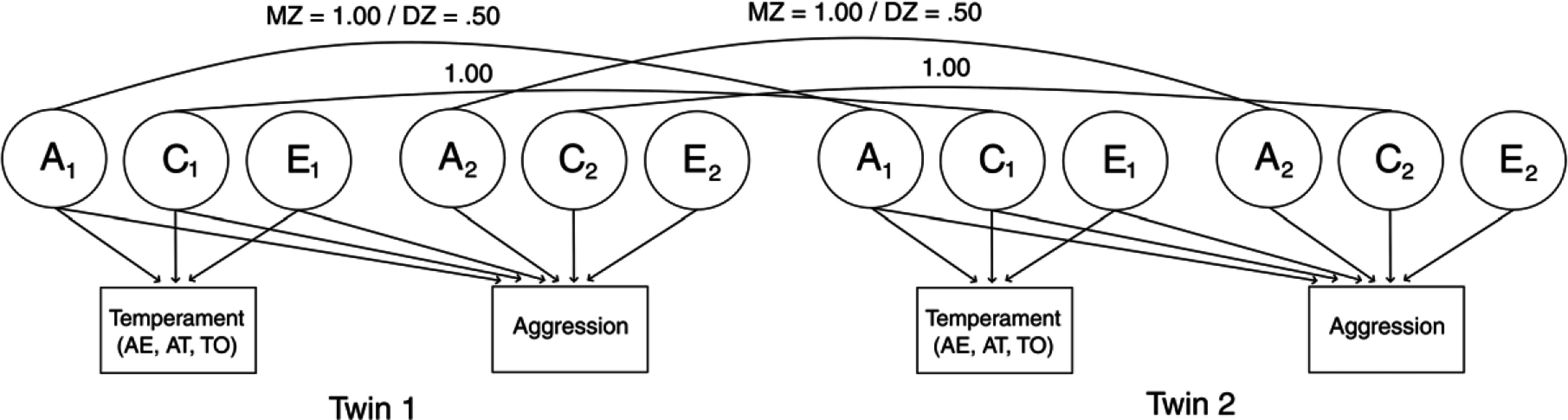
Bivariate ACE model. A = additive genetic influences; C = shared environmental influences; E = nonshared environmental influences; AT = activity; AE = affect-extraversion; TO = task orientation.

We performed bivariate biometric (ACE) model-fitting analyses using MZ and DZ variance-covariance matrices to test the etiological mechanisms underlying the association between dimensions of temperament and aggression. In a bivariate ACE model, the covariation between two traits is decomposed into genetic and environmental components, in addition to trait-specific variance decomposition. Additive genetic influences (A) delineate the cumulative effect of inherited genetic effects on a trait. Additive genetic effects are shared entirely between MZ and 50%, on average, between DZ twins. They are represented by fixing the covariance between A1 of Twin 1 and A1 of Twin 2 and 1.0 and .5 for MZ and DZ twins, respectively, in Figure 1. Shared environmental influences (C) describe the effect of experiences shared between twins raised in the same family regardless of zygosity type and is represented by fixing the covariance between C1 of Twin 1 and C1 of Twin 2 to 1.0 in Figure 1. Nonshared environmental influences (E) constitute any within-family factor that makes genetically related twins different from one another, including measurement error. These components are uncorrelated in Figure 1.

In addition to the variance decomposition of the temperament dimensions in Figure 1, there are paths connecting the ACE factors of temperament to those of aggression that quantify the covariance between them. The genetic correlation (denoted by a single-headed arrow connecting the latent A factors for each trait) indicates the extent of overlap between the genetic variances underlying each temperament dimension and aggression. Shared and nonshared environmental correlations have an analogous interpretation. Similar to how we can compute the univariate heritability, which quantifies the proportion of variance in a single trait attributed to genetic influences, we can also compute the ‘bivariate heritability’, that is, the proportion of covariance between two traits that is attributed to common genetic variance. Substantively, the bivariate heritability indicates the degree to which genetic influences explain the association between two traits, whereas the genetic correlation shows the degree to which genetic influences underlying each trait are shared (de Vries et al., 2021), providing insight into whether the traits have common (or distinct) genetic underpinnings. Shared and nonshared environmental contributions to the covariance can also be computed, with an interpretation analogous to that of the bivariate heritability.

To determine the genetic and environmental variances underlying the association between each temperament dimension and aggression, we tested three bivariate twin models: ACE, AE, and CE models. Because these models are nested, we used a chi-square difference test to compare the AE and CE models to the full ACE model. If the probability value was nonsignificant (*p* > 0.05), we omitted the shared environmental parameter (for the AE model) or the additive genetic parameter (for the CE model). Additionally, AIC, BIC, TLI, and RMSEA values were used to select the best fitting model. Once the best fitting model was identified, genetic and environmental correlations between temperament and aggression were estimated as well as genetic and environmental contributions to the phenotypic correlation.

Finally, we used bivariate sex-limitation models (Neale & Cardon, 1992) to evaluate quantitative sex differences in the association between each temperament dimension and aggression. In contrast to univariate sex-limitation models, which evaluate whether boys and girls exhibit differing magnitudes in the genetic and environmental components of a single trait, bivariate sex-limitation models allowed us to test for sex differences in the magnitudes of the genetic and environmental variance components underlying the association between each temperament dimension and aggression. We used a chi-square difference test of nested models to compare a bivariate sex-limitation model, in which the paths of A and E factors to temperament and aggression were different for boys and girls, to a model in which the paths were the same. A nonsignificant *p*-value indicated that the more parsimonious model (i.e., without quantitative sex differences) was favored.

## Results

**Figure 2.**
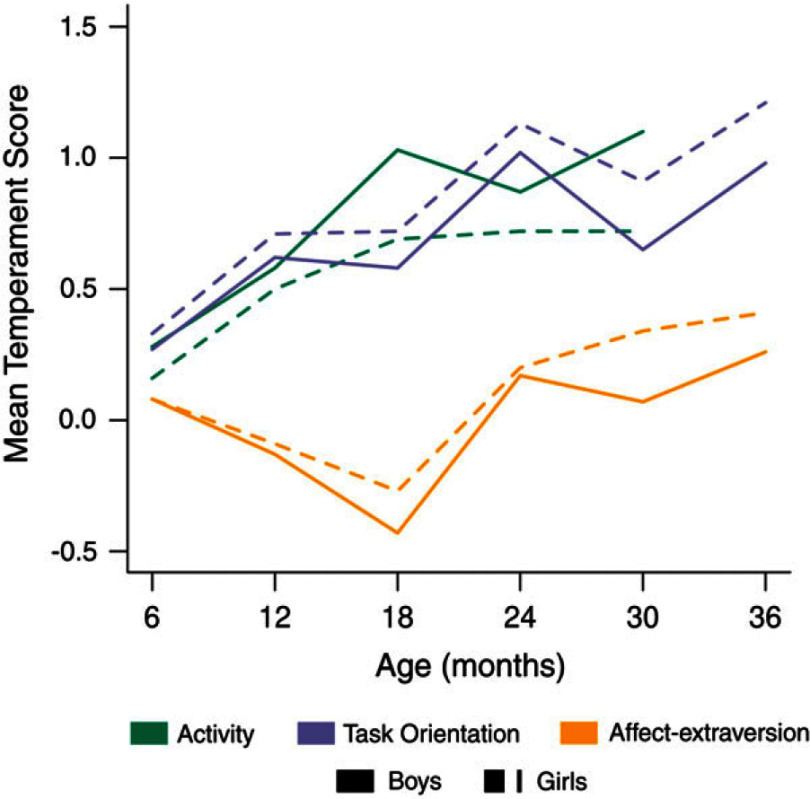
Temperament trajectories of boys and girls from 6 to 36 months.

Table 1 presents mean scores and standard deviations for each temperament dimension and aggression. Figure 2 shows mean temperament scores plotted over time. From 6 to 30 months, boys had higher mean activity scores than girls (differences ranging from .8 to .38), with significant differences at 18 months (difference: .34, *t*(495) = −4.01, *p* < .001) and 30 months (difference: .38, *t*(465) = −4.58, *p* < .001). At 6 months, boys and girls had equal mean affect-extraversion scores. From 12 to 36 months, girls exhibited higher mean affect-extraversion scores than boys (differences ranging from .03 to .27), with a significantly mean higher score at 30 months (difference: .27, *t*(457) = 3.41, *p* < .001). For task orientation, girls showed higher mean scores than boys from 6 to 36 months (differences ranging from .06 to .26), with significantly higher mean scores at 30 months (difference: .26, *t*(472) = 3.23, *p* = .001) and 36 months (difference: .23, *t*(455) = 2.70, *p* = .007). Lastly, boys showed a significantly higher mean age 7 aggression score than girls (difference: 2.3, *t*(283) = −3.03, *p* = .003).

**Table 1.** Sample sizes, means, and standard deviations for each variable at each time point

	N		Activity			Affect−Extraversion			Task Orientation			Aggression		
Age	Total	M/F	Total	Boys	Girls	Total	Boys	Girls	Total	Boys	Girls	Total	Boys	Girls
6 months	448	213/235	0.22 (0.89)	0.28 (0.93)	0.16 (0.86)	0.08 (1.08)	0.08 (1.12)	0.08 (1.05)	0.30 (0.92)	0.27 (0.91)	0.33 (0.94)	−	−	−
12 months	540	254/286	0.53 (0.89)	0.58 (0.90)	0.50 (0.88)	−0.11 (0.95)	−0.13 (0.95)	−0.09 (0.96)	0.67 (0.79)	0.62 (0.80)	0.71 (0.78)	−	−	−
18 months	512	248/264	0.86 (0.99)	1.03 (1.03)	0.69 (0.92)	−0.35 (0.98)	−0.43 (0.98)	−0.27 (0.97)	0.65 (0.87)	0.58 (0.90)	0.72 (0.83)	−	−	−
24 months	519	244/275	0.79 (0.92)	0.87 (0.93)	0.72 (0.90)	0.18 (1.01)	0.17 (1.01)	0.20 (1.02)	1.08 (0.88)	1.02 (0.88)	1.13 (0.88)	−	−	−
30 months	488	228/260	0.90 (0.95)	1.10 (0.97)	0.72 (0.89)	0.21 (0.89)	0.07 (0.93)	0.34 (0.83)	0.79 (0.89)	0.65 (0.90)	0.91 (0.87)	−	−	−
36 months	471	227/244	N/A	N/A	N/A	0.34 (0.90)	0.26 (0.91)	0.41 (0.89)	1.09 (0.92)	0.98 (0.96)	1.21 (0.87)	−	−	−
7 years	331	153/178	−	−	−	−	−	−	−	−	−	3.93 (6.86)	5.17 (7.67)	2.87 (5.90)

*Note.* Standard deviations are incorporated in parentheses. “−” indicates that data for the given variable was not collected at that time point. N/A indicates there was insufficient data for that variable to be included in the analyses. Due to the substantial time elapsed since data collection, the reason for this insufficiency is unclear.

Table 2 presents the phenotypic correlations between each temperament dimension and aggression. Correlations between activity and aggression positively increased from age 6 to 36 months, reaching a significant value of .18 at age 18 months, remaining at the same magnitude at 24 months, and increasing to a significant value of .26 at 30 months. Correlations between affect-extraversion and aggression were about zero until 24 months of age, when the correlation reached a marginally significant value of −.10, increasing to a significant value of −.20 at 30 months, then decreasing to a marginally significant value of −.12 at 36 months. Task orientation had increasingly negative correlations with aggression as the children grew older. The correlation reached a significant value of −.12 at 24 months, increased to a significant value of −.20 at 30 months, and further increased to a significant value of −.23 at 36 months. Taken together, the correlations between each temperament dimension and aggression increased over time. Notably, correlation coefficients for the associations between affect-extraversion and aggression were less stable than those of activity and task orientation.

**Table 2.** Phenotypic correlations between temperament from 6 to 36 months and aggression at age 7

	Activity	Affect−Extraversion	Task Orientation
6 months	− .01 [ − .11, .09]	− .05 [ − .16, .05]	− .05 [ − .16, .05]
12 months	.08 [ − .02, .18]	.02 [ − .09, .13]	.02 [ − .09, .13]
18 months	**.18** [.08, .29]	− .04 [ − .15, .06]	− .07 [ − .18, .04]
24 months	**.18** [.09. .28]	− .10 [ − .20, .00]	−**.12** [ − .21, −.02]
30 months	**.26** [.16, .35]	**− .20** [ − .30, −.10]	**− .20** [ − .30, −.11]
36 months	N/A	− .12 [ − .23, −.01]	**− .23** [ − .33, −.13]
Intercept	.05 [ − .05, .15]	**− .16** [ − .26, −.07]	**− .21** [ − .30, −.12]
Slope	**.23** [.15, .32]	**− .15** [ − .24, −.06]	**− .18** [ − .28, −.08]

*Note.* Values in brackets represent 95% confidence intervals. Bolded values indicate statistical significance using an alpha level of .05.

### Associations between slopes and intercepts of temperament and aggression

The latent basis growth curve model was the best fitting model for activity, affect-extraversion, and task orientation (model fit indices are presented in Table A5 of the Supplementary Materials). The intercepts for affect-extraversion and task orientation, but not activity, were significantly associated with aggression (*r* = −.16 and *r* = −.21, respectively) (Table 2). The slopes of activity, affect-extraversion, and task orientation were significantly associated with aggression, with correlation coefficients of .23, −.15, and −.18, respectively (Table 2).

### Twin correlations and cross-twin cross-trait correlations

MZ twins had greater intraclass correlations than DZ twins for each temperament dimension at each age of measurement (Table 3), with the exception of task orientation at 6 months, when the DZ correlation was about equal to the MZ correlation (.38 vs. .37, respectively). For activity, the discrepancy between MZ and DZ correlations increased from 6 to 30 months, suggesting increasing importance of genetic variance. A similar pattern was observed for affect-extraversion and task orientation, in which the difference between the MZ and DZ correlations increased from 6 to 36 months. Moreover, MZ twin correlations for both the slope and intercept of each temperament dimension were higher than those of DZ twins, suggesting genetic influences also account for individual differences in both baseline and mean-level change in temperament. Finally, MZ twins were more similar than DZ twins in aggression at age 7, suggesting that genetic influences account for variance in aggression. However, because MZ twins were not perfectly correlated on any temperament dimension or aggression, nonshared environmental variance also explains differences in each variable.

**Table 3.** Twin intraclass correlations and cross−twin cross−trait correlations

	Twin Intraclass Correlations of Individual Temperament Dimensions and Aggression			
	Activity	Affect−Extraversion	Task Orientation	Aggression
6 months	**.29** [.13, .44] / .17 [.02, .33]	**.24** [.10, .38] / **.22** [.06, .37]	**.37** [.23, .52] / **.38** [.25, .50]	−
12 months	**.30** [.15, .45] / .15 [.02, .28]	**.33** [.19, .46] / **.24** [.11, .37]	**.48** [.37, .60] / **.31** [.20, .41]	−
18 months	**.33** [.15, .51] / .14 [.02, .26]	**.31** [.18, .44] / .17 [.02, .33]	**.43** [.29, .57] / **.32** [.21, .44]	−
24 months	**.30** [.16, .45] / .06 [ − .08, .20]	**.45** [.36, .55] / .14 [ − .01, .28]	**.35** [.21, .48] / **.33** [.21, .45]	−
30 months	**.43** [.31, .55] / .07 [ − .06, .20]	**.41** [.27, .54] / .07 [ − .07, .21]	**.36** [.21, .51] / .14 [.00, .28]	−
36 months	N/A	**.61** [.52, .70] / **.27** [.12, .42]	**.63** [.53, .73] / **.35** [.21, .49]	−
7 years	−	−	−	**.73** [.55, .91] / **.42** [.20, .65]
Intercept	**.49** [.39, .60] / **.28** [.16, .42]	**.69** [.63, .76] / **.36** [.25, .48]	**.86** [.81, .89] / **.71** [.65, .77]	−
Slope	**.46** [.34, .58] / **.17** [.03, .31]	**.74** [.68, .80] / **.72** [.66, .77]	**.79** [.73, .85] / **.65** [.58, .72]	−

	Cross−twin Cross−Trait Correlations between Temperament Dimensions and Aggression			
	Activity with Aggression	Affect−Extraversion with Aggression	Task Orientation with Aggression	
6 months	− .01 [ − .15, .13] / −.10 [ − .26, .05]	− .08 [ − .17, .01] / −.10 [ − .19, −.01]	− .05 [ − .20, .10] / −**.14** [ − .25, −.03]	
12 months	.12 [ − .01, .24] / .05 [ − .09, .18]	.00 [ − .13, .13] / −.03 [ − .14, .07]	.01 [ − .12, .14] / −.02 [ − .14, .10]	
18 months	.13 [.01, .24] / **.16** [.08, .23]	− .07 [ − .17, .02] / **− .13** [ − .23, −.03]	− .08 [ − .24, .07] / −.04 [ − .15, .06]	
24 months	**.24** [.15, .34] / −.01 [ − .12, .09]	**− .11** [ − .23, .00] / **− .12** [ − .21, −.03]	**− .16** [ − .28, −.03] / −.06 [ − .17, .04]	
30 months	**.19** [.08, .31] / .13 [ − .01, .27]	**− .18** [ − .30, −.07] / −.05 [ − .15, .05]	**− .20** [ − .30, −.10] / −.04 [ − .17, .08]	
36 months	N/A	**− .15** [ − .25, −.06] / −.01 [ − .14, .13]	**− .24** [ − .35, −.12] / −.08 [ − .23, .07]	
Intercept	.05 [ − .11, .20] / −.11 [ − .27, .06]	**− .22** [ − .31, −.13] / **− .14** [ − .24, −.05]	**− .13** [ − .22, −.03] / −.01 [ − .12, .09]	
Slope	**.22** [.09, .36] / **.17** [.03, .31]	**− .25** [ − .37, −.13] / **− .19** [ − .30, −.07]	**− .34** [ − .47, −.21] / **− .23** [ − .35, −.11]	

*Note.* Values in brackets represent 95% confidence intervals. Bolded values indicate statistical significance using an alpha level of .05. Correlations above are unadjusted for sex and socioeconomic status. Correlations are denoted in MZ / DZ format.

For cross-twin cross-trait associations, no discernible trend was observed until about 24 months, when correlations of activity and task orientation with aggression for MZ twins became significant and reliably larger than those of DZ twins (Table 3). MZ correlations between affect-extraversion and aggression became reliably larger than DZ correlations at 30 months. The correlations of the intercept and slope of each temperament dimension with aggression were generally greater in MZ than in DZ twins, with the exception of the association between the intercept of activity and aggression, in which MZ and DZ correlations were both small and nonsignificant. Like the univariate twin correlations presented above, these findings suggest that genetic variance underlie the association between each temperament dimension and aggression.

### Model-fitting results for biometric bivariate models

The bivariate AE model, in which the C (shared environment) parameter was dropped from the original ACE model, was the best fitting model for the association between each temperament dimension and aggression (Tables 4–6). The CE model, postulating no additive genetic influences underlying the association between each temperament dimension and aggression, fitted worse than the AE model.

**Table 4.** Model fit statistics for bivariate ACE models examining the association between activity and aggression

Activity	Model	df	χ2	AIC	BIC	RMSEA	TLI	χ2 _diff_	df _diff_	*p*
6 months	ACE	43	25.84	3310.39	3371.31	.00	1.00	–	–	–
	**AE**	**46**	**27.14**	**3305.69**	**3355.86**	**.00**	**1.00**	**1.30**	**3.00**	**.73**
	CE	46	37.65	3316.20	3366.37	.00	1.00	11.81	3.00	.01
12 months	ACE	43	24.72	3534.14	3596.29	.00	1.00	–	–	–
	**AE**	**46**	**24.77**	**3528.19**	**3579.37**	**.00**	**1.00**	**0.05**	**3.00**	**1.00**
	CE	46	38.96	3542.38	3593.56	.00	1.00	14.24	3.00	.00
18 months	ACE	43	67.84	3570.03	3631.45	.07	.74	–	–	–
	**AE**	**46**	**68.54**	**3564.73**	**3615.31**	**.06**	**0.78**	**0.70**	**3.00**	**.87**
	CE	46	79.86	3576.05	3626.63	.07	.67	12.02	3.00	.01
24 months	ACE	43	34.16	3505.76	3566.87	.00	1.00	–	–	–
	**AE**	**46**	**34.99**	**3500.60**	**3550.92**	**.00**	**1.00**	**0.84**	**3.00**	**.84**
	CE	46	51.16	3516.76	3567.09	.03	.94	17.00	3.00	.00
30 months	ACE	43	45.20	3449.21	3509.47	.02	.98	–	–	–
	**AE**	**46**	**45.26**	**3443.27**	**3492.90**	**.00**	**1.00**	**0.06**	**3.00**	**1.00**
	CE	46	62.16	3460.16	3509.80	.05	.85	16.96	3.00	.00
Intercept	ACE	43	43.03	2104.48	2167.56	.00	1.00	–	–	–
	**AE**	**46**	**44.37**	**2099.82**	**2151.76**	**.00**	**1.00**	**1.33**	**3.00**	**.72**
	CE	46	57.60	2113.05	2164.99	.04	.94	14.56	3.00	.00
Slope	ACE	43	65.23	2703.47	2766.55	.06	.84	–	–	–
	**AE**	**46**	**65.48**	**2697.72**	**2749.66**	**.05**	**.87**	**0.25**	**3.00**	**.97**
	CE	46	80.07	2712.31	2764.25	.07	.77	14.84	3.00	.00

*Note.* The model in bold represents the best fitting model. df = degrees of freedom; χ2 = chi-square; AIC = Akaike information criterion; BIC = Bayesian information criterion, RMSEA = Root Mean Square Error of Approximation; TLI = Tucker Lewis Index.

**Table 5.** Model fit statistics for bivariate ACE models examining the association between affect-extraversion and aggression

Affect-Extraversion	Model	df	χ2	AIC	BIC	RMSEA	TLI	χ2 _diff_	df _diff_	*p*
6 months	ACE	43	32.02	3465.28	3526.20	.00	1.00	–	–	–
	**AE**	**46**	**32.94**	**3460.19**	**3510.36**	**.00**	**1.00**	**0.91**	**3.00**	**.82**
	CE	46	43.91	3471.16	3521.33	.00	1.00	11.88	3.00	.01
12 months	ACE	43	42.91	3599.95	3662.10	.00	1.00	–	–	–
	**AE**	**46**	**44.40**	**3595.44**	**3646.63**	**.00**	**1.00**	**1.49**	**3.00**	**.69**
	CE	46	56.06	3607.10	3658.28	.04	.90	13.14	3.00	.00
18 months	ACE	43	34.33	3553.93	3615.35	.00	1.00	–	–	–
	**AE**	**46**	**34.94**	**3548.54**	**3599.12**	**.00**	**1.00**	**0.61**	**3.00**	**.90**
	CE	46	47.99	3561.59	3612.18	.02	.98	13.66	3.00	.00
24 months	ACE	43	25.98	3590.34	3651.45	.00	1.00	–	–	–
	**AE**	**46**	**26.15**	**3584.50**	**3634.83**	**.00**	**1.00**	**0.16**	**3.00**	**.98**
	CE	46	45.61	3603.97	3654.30	.00	1.00	19.63	3.00	.00
30 months	ACE	43	37.21	3386.05	3446.32	.00	1.00	–	–	–
	**AE**	**46**	**37.37**	**3380.21**	**3429.84**	**.00**	**1.00**	**0.16**	**3.00**	**.98**
	CE	46	55.09	3397.93	3447.56	.04	.91	17.88	3.00	.00
36 months	ACE	43	35.13	3313.80	3373.11	.00	1.00	–	–	–
	**AE**	**46**	**35.79**	**3308.45**	**3357.30**	**.00**	**1.00**	**0.66**	**3.00**	**.88**
	CE	46	60.46	3333.13	3381.97	.05	.90	25.33	3.00	.00
Intercept	ACE	43	63.93	2237.77	2300.96	.06	.92	–	–	–
	**AE**	**46**	**63.98**	**2231.81**	**2283.85**	**.05**	**.93**	**0.05**	**3.00**	**1.00**
	CE	46	98.06	2265.90	2317.93	.09	.81	34.13	3.00	.00
Slope	ACE	43	43.48	−159.60	−96.41	.01	1.00	–	–	–
	**AE**	**46**	**45.59**	**−163.49**	**−111.45**	**.00**	**1.00**	**2.11**	**3.00**	**.55**
	CE	46	57.15	−151.94	−99.90	.04	.99	13.67	3.00	.00

*Note.* The model in bold represents the best fitting model. df = degrees of freedom; χ2 = chi-square; AIC = Akaike information criterion; BIC = Bayesian information criterion, RMSEA = Root Mean Square Error of Approximation; TLI = Tucker Lewis Index.

**Table 6.** Model fit statistics for bivariate ACE models examining the association between task orientation and aggression

Task Orientation	Model	df	χ2	AIC	BIC	RMSEA	TLI	χ2 _diff_	df _diff_	*p*
6 months	ACE	43	31.44	3310.48	3371.40	.00	1.00	–	–	–
	**AE**	**46**	**37.44**	**3310.49**	**3360.66**	**.00**	**1.00**	**6.01**	**3.00**	**.11**
	CE	46	43.67	3316.71	3366.88	.00	1.00	12.23	3.00	.01
12 months	ACE	43	33.24	3332.82	3394.97	.00	1.00	–	–	–
	**AE**	**46**	**33.43**	**3327.01**	**3378.20**	**.00**	**1.00**	**0.20**	**3.00**	**.98**
	CE	46	49.23	3342.81	3393.99	.02	.98	15.99	3.00	.00
18 months	ACE	43	42.20	3384.64	3446.07	.00	1.00	–	–	–
	**AE**	**46**	**42.87**	**3379.31**	**3429.90**	**.00**	**1.00**	**0.67**	**3.00**	**.88**
	CE	46	56.07	3392.51	3443.09	.04	.93	13.87	3.00	.00
24 months	ACE	43	33.78	3427.06	3488.17	.00	1.00	–	–	–
	**AE**	**46**	**35.00**	**3422.28**	**3472.60**	**.00**	**1.00**	**1.22**	**3.00**	**.75**
	CE	46	47.20	3434.48	3484.80	.01	.99	13.42	3.00	.00
30 months	ACE	43	24.29	3380.25	3440.52	.00	1.00	–	–	–
	**AE**	**46**	**24.72**	**3374.68**	**3424.31**	**.00**	**1.00**	**0.43**	**3.00**	**.93**
	CE	46	40.14	3390.10	3439.73	.00	1.00	15.85	3.00	.00
36 months	ACE	43	35.58	3303.41	3362.72	.00	1.00	–	–	–
	**AE**	**46**	**36.20**	**3298.02**	**3346.87**	**.00**	**1.00**	**0.62**	**3.00**	**.89**
	CE	46	59.17	3321.00	3369.84	.05	.93	23.59	3.00	.00
Intercept	ACE	43	77.27	1754.09	1817.28	.07	.95	–	–	–
	**AE**	**46**	**79.17**	**1749.99**	**1802.03**	**.07**	**.95**	**1.91**	**3.00**	**.59**
	CE	46	104.65	1775.47	1827.50	.09	.92	27.38	3.00	.00
Slope	ACE	43	64.69	1582.00	1645.19	.06	.96	–	–	–
	**AE**	**46**	**67.55**	**1578.86**	**1630.89**	**.06**	**.96**	**2.85**	**3.00**	**.42**
	CE	46	87.41	1598.72	1650.76	.08	.92	22.72	3.00	.00

*Note.* The model in bold represents the best fitting model. df = degrees of freedom; χ2 = chi-square; AIC = Akaike information criterion; BIC = Bayesian information criterion, RMSEA = Root Mean Square Error of Approximation; TLI = Tucker Lewis Index.

**Figure 3.**
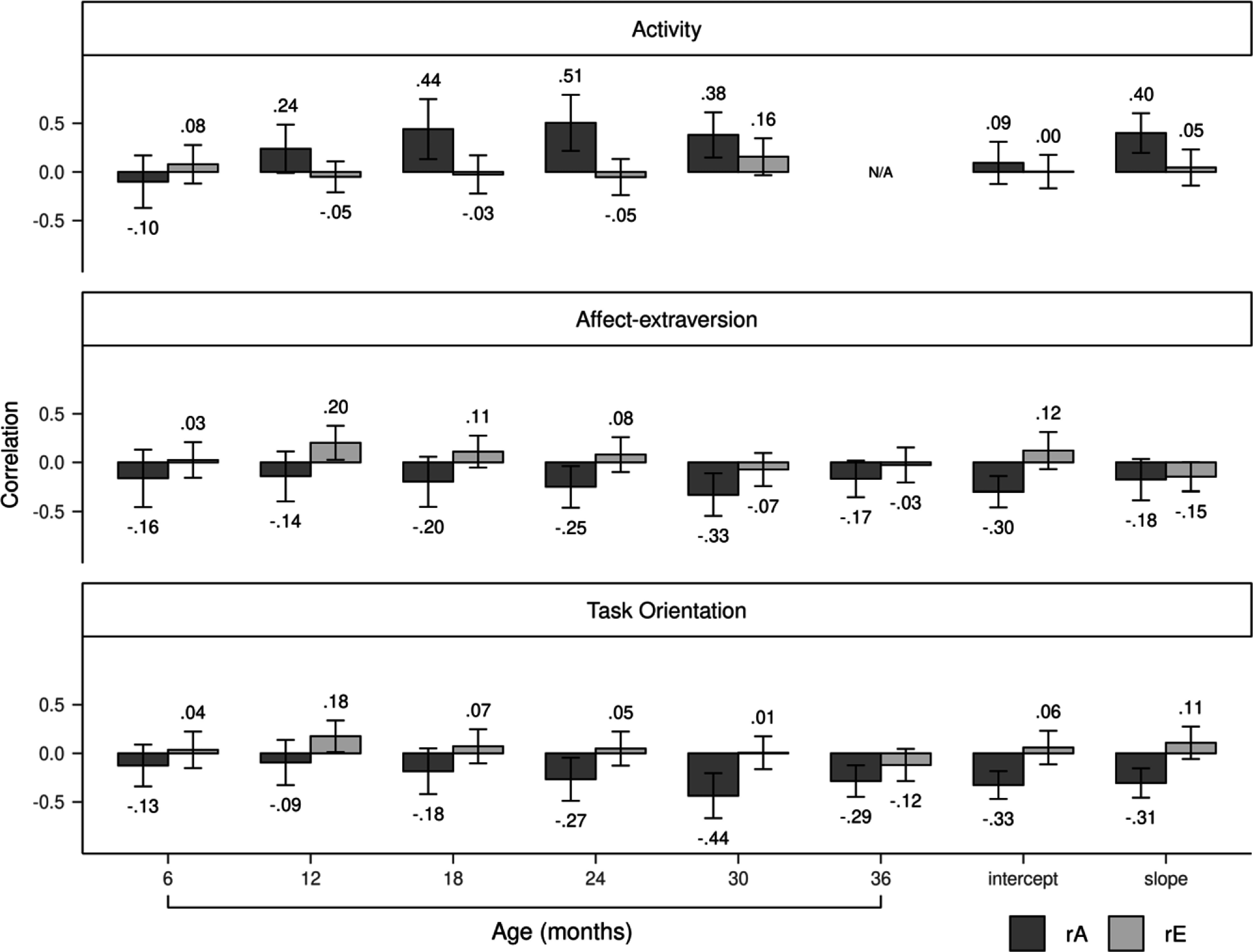
Genetic and nonshared environmental correlations of the three temperament dimensions with aggression. Error bars denote 95% confidence intervals. rA = genetic correlation; rE = nonshared environmental correlation.

**Figure 4.**
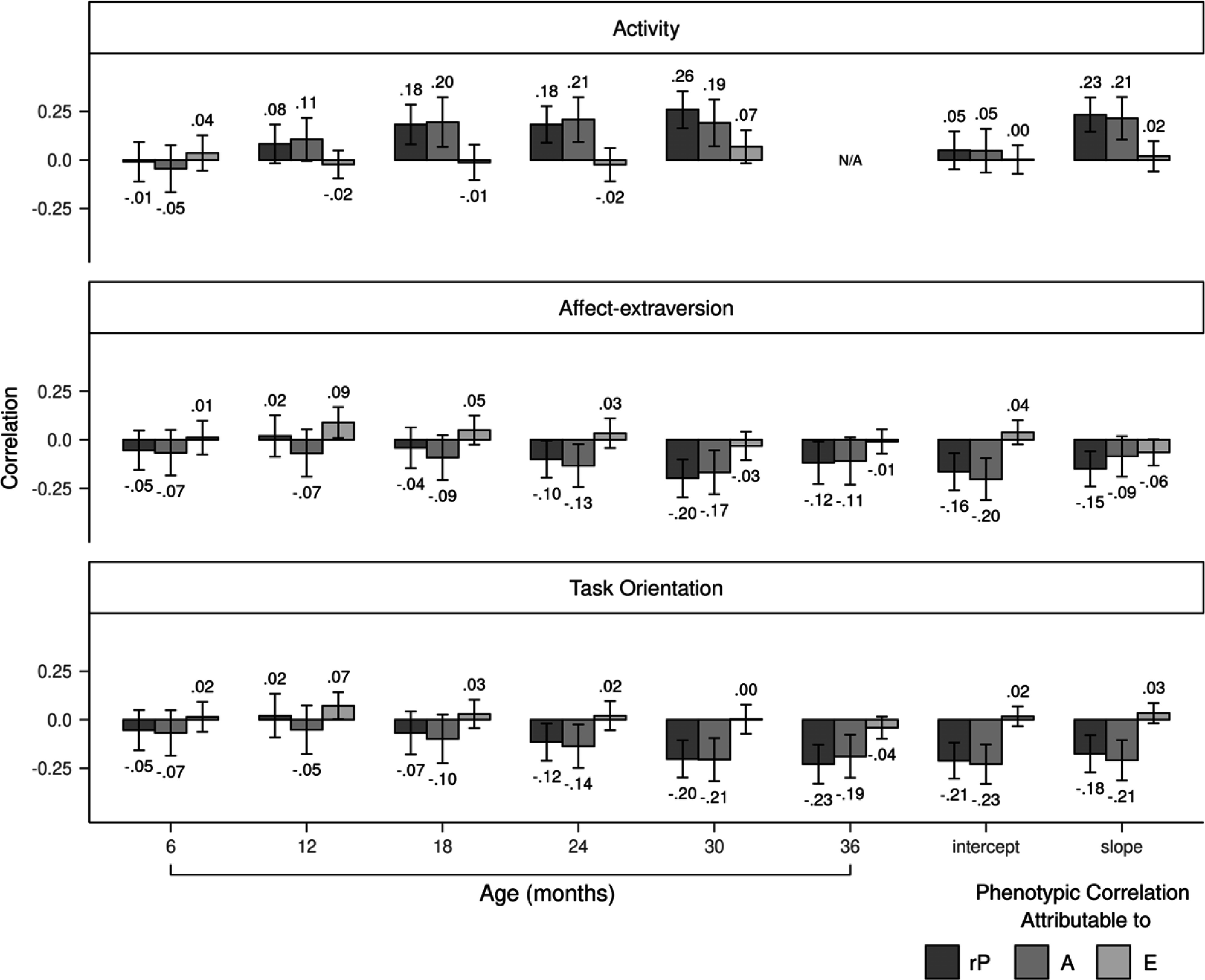
Genetic and nonshared environmental contributions to the covariation between temperament dimensions and aggression. Error bars denote 95% confidence intervals. rP = phenotypic correlation; A = additive genetic influences; E = nonshared environmental influences.

Genetic correlations between activity and aggression positively increased from 6 to 30 months, peaking at a significant value of .51 at 24 months, before decreasing to a smaller but still significant value of .38 at 30 months (Figure 3). The slope of activity, but not the intercept, was genetically correlated to aggression (*r* = .40). Nonshared environmental correlations tended to be small and nonsignificant from 6 to 30 months. Genetic influences completely accounted for the phenotypic correlations between activity and aggression from 6 to 24 months (Figure 4). By 30 months, genetic influences explained 74% of the correlation, with nonshared environmental influences accounting for the remaining 26%. Genetic and nonshared environmental influences explained 92% and 8% of the correlation, respectively, between the slope of activity and aggression.

Genetic correlations between affect-extraversion and aggression negatively increased from 6 to 36 months (Figure 3). The genetic correlation reached a significant value of −.25 at 24 months, increased to a significant value of −.33 at 30 months, and decreased to a nonsignificant value of −.17 at 36 months. The intercept and slope had negative genetic correlations with aggression, with values of −.30 and −.18, respectively, but only the former was significant. Nonshared environmental correlations were unstable from 6 to 36 months. The slope of affect-extraversion and aggression had a nonshared environmental correlation of −.15 but was not significant. Genetic influences fully explained the phenotypic correlations between affect-extraversion and aggression from 6 to 24 months (Figure 4). At 30 months, genetic influences accounted for 84% of the phenotypic correlation, with the rest explained by nonshared environmental influences. This effect diminished slightly at 36 months, when genetic and nonshared environmental influences explained 92% and 8% of the phenotypic correlation, respectively. Genetic and nonshared environmental influences each explained about half of the phenotypic correlation between the slope of affect-extraversion and aggression.

Genetic correlations between task orientation and aggression negatively increased from 6 to 36 months (Figure 3). Genetic correlations reached a significant value of −.27 at 24 months, increased to a significant value of −.44 at 30 months, and decreased to a smaller but still significant value of −.29 at age 36 months. Both the intercept and slope of task orientation had significant genetic correlations with aggression, with values of −.33 and −.31, respectively. Nonshared environmental correlations with aggression were nonsignificant for both the slope and intercept of task orientation. Nonshared environmental correlations between task orientation and aggression were unstable from 6 to 36 months. Genetic influences fully explained the phenotypic correlations between task orientation and aggression from 6 to 30 months (Figure 4). At 36 months, genetic and nonshared environmental influences explained 82% and 18% of the phenotypic correlation, respectively. Genetic influences entirely explained the phenotypic correlations between aggression and the intercept and slope of task orientation.

### Exploratory analysis of sex differences

Quantitative sex differences were found for the associations between each temperament dimension and aggression. Bivariate AE models with varying parameter estimates for boys and girls fit better than models with parameter estimates equated across sex (Tables 7–9).

**Table 7.** Sex-limitation models for activity and aggression

Activity	Model	df	χ2	AIC	BIC	RMSEA	TLI	χ2 _diff_	df _diff_	*p*
6 months	**AE with sex diff**	**109**	**158.20**	**3282.63**	**3357.88**	**.09**	**.47**	–	–	–
	AE no sex diff	115	174.29	3286.72	3340.47	.10	.39	16.09	6.00	.01
12 months	**AE with sex diff**	**109**	**132.41**	**3494.87**	**3571.65**	**.06**	**.74**	–	–	–
	AE no sex diff	115	153.76	3504.23	3559.07	.08	.59	21.36	6.00	.00
18 months	**AE with sex diff**	**109**	**174.12**	**3540.03**	**3615.90**	**.10**	**.28**	–	–	–
	AE no sex diff	115	194.23	3548.14	3602.34	.11	.17	20.11	6.00	.00
24 months	**AE with sex diff**	**109**	**158.81**	**3475.62**	**3551.11**	**.09**	**.56**	–	–	–
	AE no sex diff	115	178.77	3483.58	3537.50	.10	.47	19.96	6.00	.00
30 months	**AE with sex diff**	**109**	**133.38**	**3412.40**	**3486.85**	**.07**	**.69**	–	–	–
	AE no sex diff	115	155.88	3422.90	3476.08	.08	.51	22.50	6.00	.00
Intercept	**AE with sex diff**	**109**	**180.95**	**2080.55**	**2158.47**	**.11**	**.70**	–	–	–
	AE no sex diff	115	202.57	2090.17	2145.83	.11	.66	21.62	6.00	.00
Slope	**AE with sex diff**	**109**	**173.95**	**2668.49**	**2746.41**	**.10**	**.51**	–	–	–
	AE no sex diff	115	199.04	2681.57	2737.23	.11	.40	25.08	6.00	.00

*Note.* The model in bold represents the best fitting model. df = degrees of freedom; χ2 = chi-square; AIC = Akaike information criterion; BIC = Bayesian information criterion, RMSEA = Root Mean Square Error of Approximation; TLI = Tucker Lewis Index.

**Table 8.** Sex-limitation models for affect-extraversion and aggression

Affect-Extraversion	Model	df	χ2	AIC	BIC	RMSEA	TLI	χ2 _diff_	df _diff_	*p*
6 months	**AE with sex diff**	**109**	**131.36**	**3433.67**	**3508.92**	**.06**	**.76**	–	–	–
	AE no sex diff	115	147.86	3438.17	3491.92	.07	.66	16.50	6.00	.01
12 months	**AE with sex diff**	**109**	**142.75**	**3559.88**	**3636.66**	**.07**	**.67**	–	–	–
	AE no sex diff	115	165.44	3570.57	3625.41	.09	.54	22.69	6.00	.00
18 months	**AE with sex diff**	**109**	**159.08**	**3508.85**	**3584.73**	**.09**	**.62**	–	–	–
	AE no sex diff	115	192.44	3530.22	3584.42	.11	.45	33.37	6.00	.00
24 months	**AE with sex diff**	**109**	**136.09**	**3557.65**	**3633.14**	**.07**	**.79**	–	–	–
	AE no sex diff	115	157.16	3566.73	3620.65	.08	.69	21.08	6.00	.00
30 months	**AE with sex diff**	**109**	**151.54**	**3351.55**	**3426.00**	**.09**	**.54**	–	–	–
	AE no sex diff	115	172.21	3360.22	3413.40	.10	.42	20.67	6.00	.00
36 months	**AE with sex diff**	**109**	**140.20**	**3277.95**	**3351.22**	**.08**	**.80**	–	–	–
	AE no sex diff	115	163.50	3289.26	3341.59	.09	.71	23.30	6.00	.00
Intercept	**AE with sex diff**	**109**	**178.57**	**2206.85**	**2284.91**	**.10**	**.75**	–	–	–
	AE no sex diff	115	201.74	2218.03	2273.78	.11	.70	23.17	6.00	.00
Slope	**AE with sex diff**	**109**	**161.05**	**−194.05**	**−116.00**	**.09**	**.94**	–	–	–
	AE no sex diff	115	188.46	−178.64	−122.89	.10	.92	27.41	6.00	.00

*Note.* The model in bold represents the best fitting model. df = degrees of freedom; χ2 = chi-square; AIC = Akaike information criterion; BIC = Bayesian information criterion, RMSEA = Root Mean Square Error of Approximation; TLI = Tucker Lewis Index.

**Table 9.** Sex-limitation models for task orientation and aggression

Task Orientation	Model	df	χ2	AIC	BIC	RMSEA	TLI	χ2 _diff_	df _diff_	*p*
6 months	**AE with sex diff**	**109**	**168.70**	**3274.71**	**3349.96**	**.10**	**.60**	–	–	–
	AE no sex diff	115	203.47	3297.48	3351.23	.12	.44	34.77	6.00	.00
12 months	**AE with sex diff**	**109**	**127.15**	**3291.77**	**3368.54**	**.05**	**.89**	–	–	–
	AE no sex diff	115	150.02	3302.63	3357.47	.07	.80	22.86	6.00	.00
18 months	**AE with sex diff**	**109**	**158.64**	**3348.19**	**3424.07**	**.09**	**.73**	–	–	–
	AE no sex diff	115	181.77	3359.32	3413.52	.10	.65	23.13	6.00	.00
24 months	**AE with sex diff**	**109**	**140.13**	**3398.39**	**3473.88**	**.07**	**.77**	–	–	–
	AE no sex diff	115	160.50	3406.77	3460.69	.09	.68	20.38	6.00	.00
30 months	**AE with sex diff**	**109**	**137.30**	**3349.47**	**3423.92**	**.07**	**.74**	–	–	–
	AE no sex diff	115	156.50	3356.66	3409.84	.08	.63	19.20	6.00	.00
36 months	**AE with sex diff**	**109**	**150.33**	**3273.13**	**3346.40**	**.09**	**.80**	–	–	–
	AE no sex diff	115	177.11	3287.91	3340.24	.11	.72	26.78	6.00	.00
Intercept	**AE with sex diff**	**109**	**167.84**	**1741.79**	**1819.85**	**.09**	**.92**	–	–	–
	AE no sex diff	115	188.18	1750.14	1805.89	.10	.91	20.35	6.00	.00
Slope	**AE with sex diff**	**109**	**156.67**	**1561.38**	**1639.43**	**.09**	**.91**	–	–	–
	AE no sex diff	115	178.04	1570.75	1626.51	.10	.89	21.38	6.00	.00

*Note.* The model in bold represents the best fitting model. df = degrees of freedom; χ2 = chi-square; AIC = Akaike information criterion; BIC = Bayesian information criterion, RMSEA = Root Mean Square Error of Approximation; TLI = Tucker Lewis Index.

**Figure 5.**
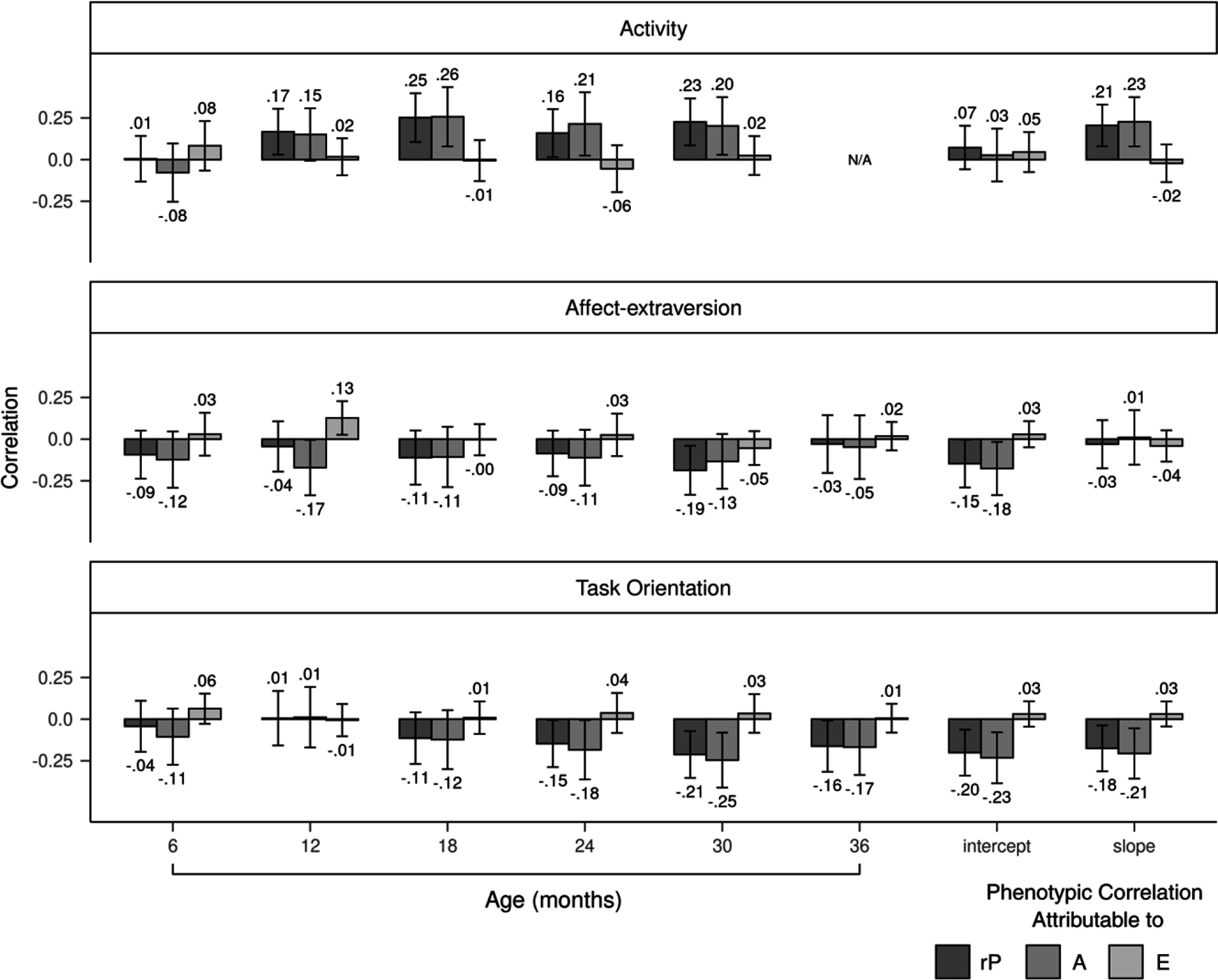
Genetic and nonshared environmental contributions to the covariation between temperament dimensions and aggression for boys. Error bars denote 95% confidence intervals. rP = phenotypic correlation; A = additive genetic influences; E = nonshared environmental influences.

**Figure 6.**
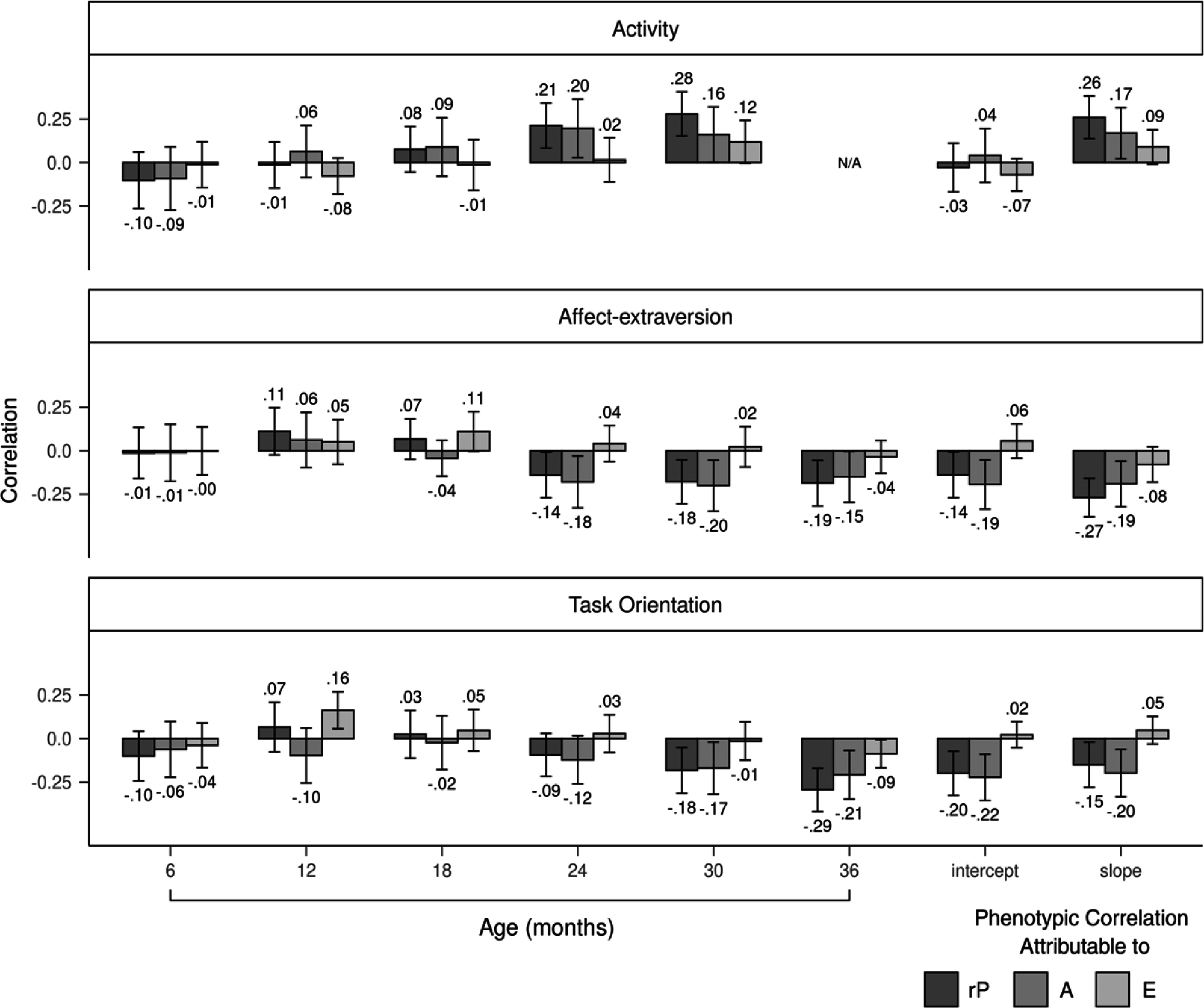
Genetic and nonshared environmental contributions to the covariation between temperament dimensions and aggression for girls. Error bars denote 95% confidence intervals. rP = phenotypic correlation; A = additive genetic influences; E = nonshared environmental influences.

**Figure 7.**
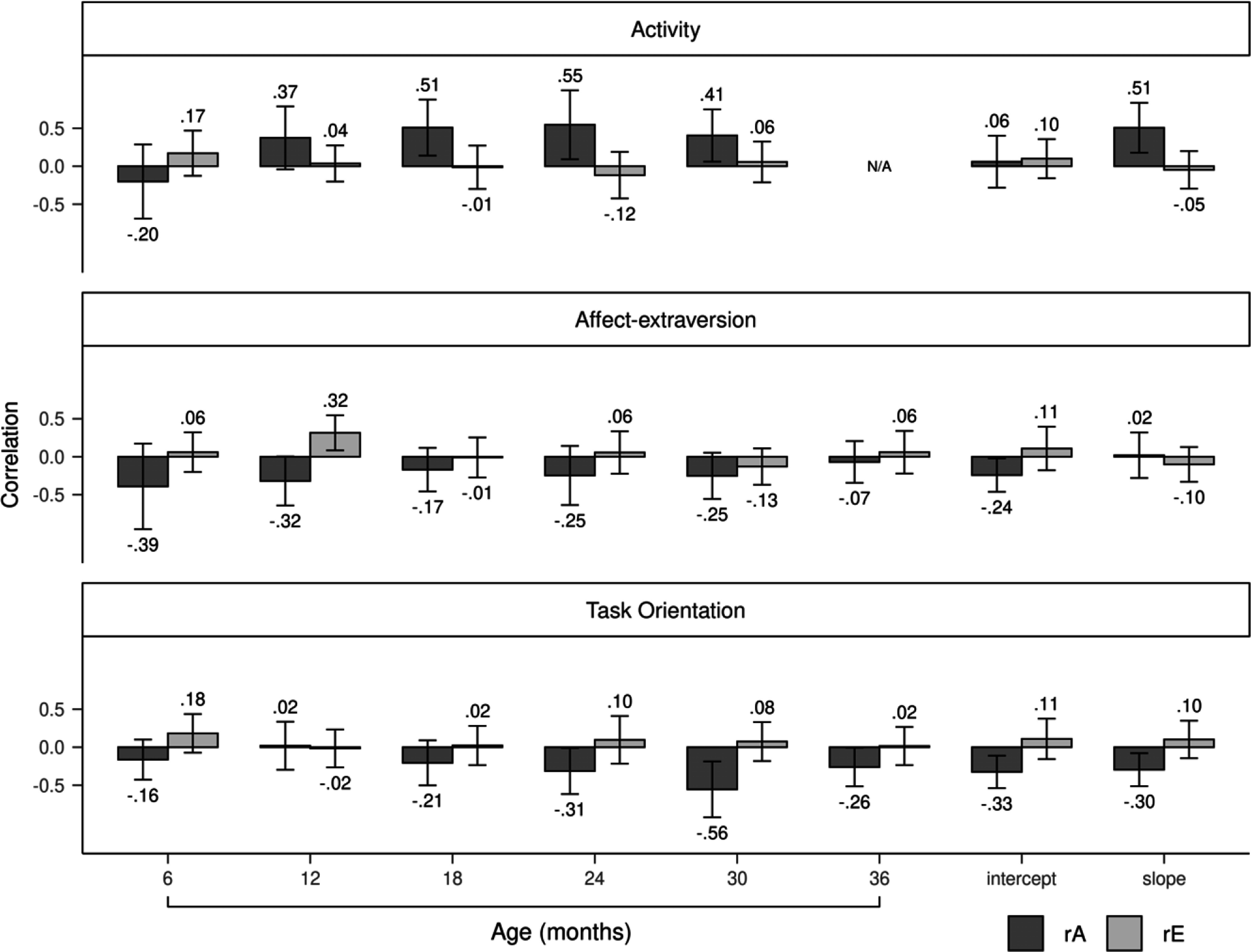
Genetic and nonshared environmental correlations of the three temperament dimensions with aggression for boys. Error bars denote 95% confidence intervals. rA = genetic correlation; rE = nonshared environmental correlation.

**Figure 8.**
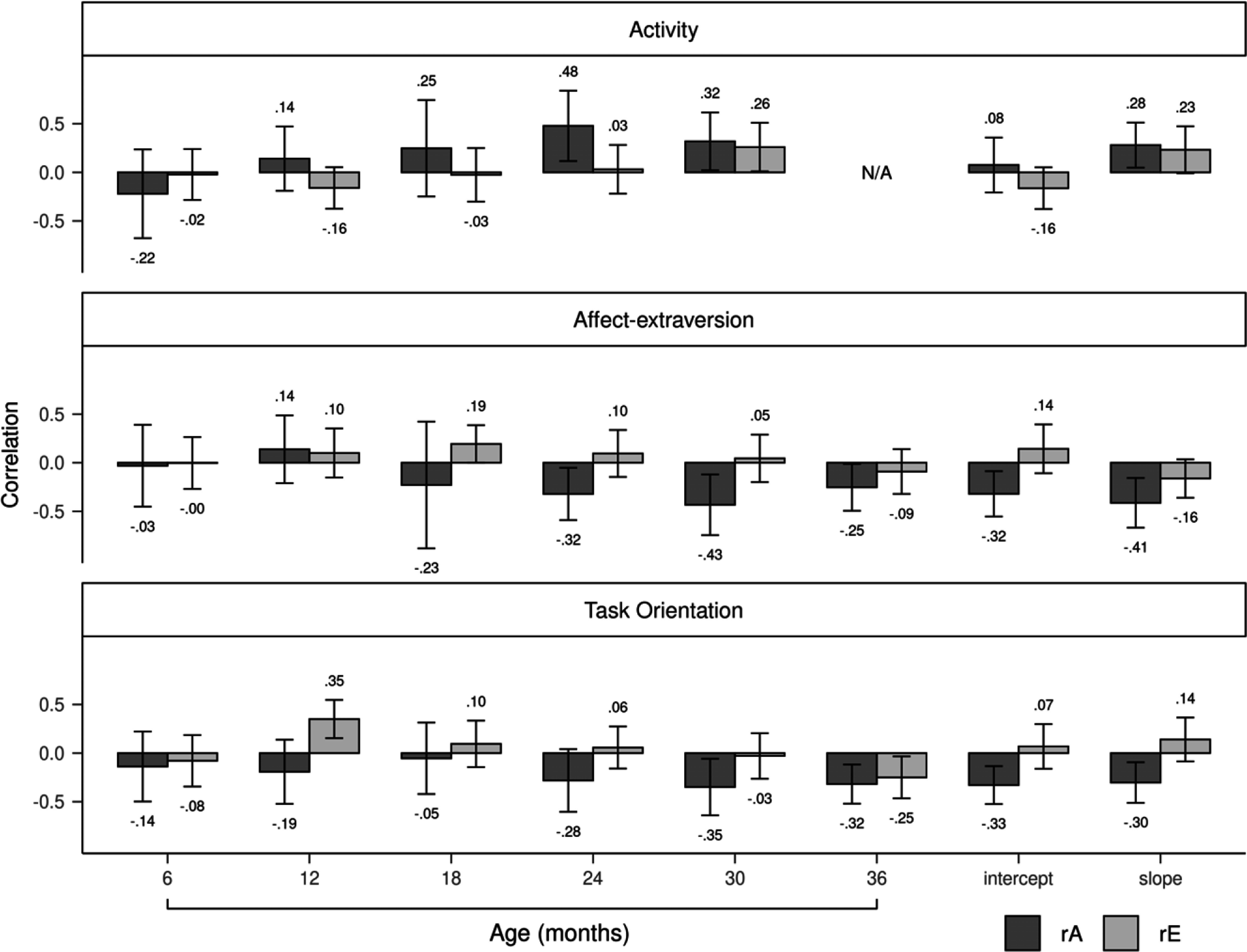
Genetic and nonshared environmental correlations of the three temperament dimensions with aggression for girls. Error bars denote 95% confidence intervals. rA = genetic correlation; rE = nonshared environmental correlation.

Phenotypic correlations between activity and aggression positively increased for boys (Figure 5) earlier than girls (Figure 6). For boys, by 12 months, the correlation between activity and aggression reached a significant value of .17 and continued to positively increase thereafter. By contrast, for girls, the phenotypic correlation first became significant at 24 months with a value of .21. Genetic influences primarily accounted for the phenotypic correlations between activity and aggression for boys and girls. At 30 months, however, nonshared environmental influences accounted for a greater proportion of the phenotypic correlation for girls than boys (43% for girls vs. 9% for boys). Nonshared environmental influences accounted for 35% of the correlation between the slope of activity and aggression for girls, whereas for boys, nonshared environmental influences accounted for none of the correlation. Genetic correlations between activity and aggression were generally larger for boys (Figure 7) than girls (Figure 8). Girls, but not boys, exhibited a positive but nonsignificant nonshared environmental correlation between the slope of activity and aggression (.23 for girls vs. −.05 for boys).

Phenotypic correlations between affect-extraversion and aggression were negative from 6 to 36 months for boys (Figure 5). For girls (Figure 6), the correlation only became negative at 24 months when it reached a marginally significant value of −.14 and continued to negatively increase thereafter. Girls, but not boys, had a significant negative correlation between the slope of affect-extraversion and aggression (*r* = −.27), with 71% of this correlation explained by genetic influences and the remaining 29% explained by nonshared environmental influences. Genetic influences entirely accounted for the phenotypic correlations from 6 to 24 months for boys. At 30 months, however, nonshared environmental influences accounted for 26% of the phenotypic correlation for boys, and at 36 months, nonshared environmental influences accounted for 21% of the phenotypic correlation for girls. Genetic correlations between affect-extraversion and aggression both trended in the negative direction from 6 to 36 months for boys (Figure 7) and girls (Figure 8), but only girls had significant genetic correlations. Likewise, the intercept and slope of affect-extraversion were genetically correlated with aggression in girls but not boys, with correlation coefficients of −.32 and −.41, respectively.

Phenotypic correlations between task orientation and aggression were generally larger for boys (Figure 5) than girls (Figure 6). For boys, nonshared environmental contributions to the phenotypic correlations were null. For girls, at 30 and 36 months, nonshared environmental influences began to account for 6% and 30% of the phenotypic correlation, respectively. For boys (Figure 7) and girls (Figure 8), genetic correlations negatively increased, reaching peak values of −.56 and −.35 at 30 months, respectively. Nonshared environmental correlations were unstable for boys and girls.

## Discussion

The present study elucidated the temporal associations between infant and toddler temperament and childhood aggression as well as their underlying genetic and environmental etiologies. In line with our first hypothesis, we found that the correlations between early-life temperament and aggression at age 7 increased from 6 to 36 months. Activity was the first temperament dimension to become correlated with aggression at 18 months, followed by task orientation at 24 months, and affect-extraversion at 30 months. Latent growth curve models revealed that greater rates of change in each temperament dimension correlated as expected with higher (for activity) and lower (for task orientation and affect-extraversion) levels of aggression. Only baseline levels (i.e., intercepts) of affect-extraversion and task orientation, but not activity, explained individual differences in aggression. In keeping with a spectrum conceptualization of temperament and aggression, genetically informed models revealed that common genetic variance primarily explained associations between each temperament dimension and aggression, with evidence of nonshared environmental influences beginning to account for a small but nonsignificant proportion of the correlation by 36 months. Moreover, genetic variance underlying temperament and aggression remained largely unique, despite genetic influences becoming increasingly common throughout development.

### Activity

As hypothesized, phenotypic correlations between infant and toddler activity and aggression at age 7 years positively increased from 6 to 36 months. Associations were negligible in the first year of life but became significant at 18 months. Furthermore, those increasing in activity at faster rates were more likely to be aggressive at age 7 years regardless of their baseline levels of activity. These findings are consistent with prior studies that have found significant positive associations between activity and aggression (Nwadinobi & Gagne, 2020) and broader externalizing behavior problems (Fagot & O’Brien, 1994; Scheper et al., 2017; Teglasi & MacMahon, 1990). Two longitudinal studies (Lahey et al., 2008; Morales et al., 2022) found significant associations between activity and childhood externalizing outcomes at earlier time points (4 and 6 months, respectively) than the present study (18 months). It may be the case that associations between activity and broader externalizing outcomes, including aggression and attention problems in one study (Morales et al., 2022), emerge earlier in life than specific associations with aggression. Alternatively, there may be sample-to-sample variability in the age at which activity and childhood aggression begin to correlate with one another.

Genetic correlations between activity and aggression increased throughout infancy and toddlerhood, suggesting that a child’s genetic propensity for both activity and aggression becomes more pronounced throughout development. Moreover, common genetic influences almost entirely explained the phenotypic correlations between activity and aggression, further supporting a spectrum conceptualization of both traits. Interestingly, nonshared environmental influences began to contribute to the correlation by 30 months, though this effect was small and nonsignificant. Together, these findings are broadly consistent with studies that have reported significant genetic overlap between temperamental activity and ADHD/externalizing symptoms (Ilott et al., 2010; Saudino et al., 2018). It should be noted, however, that neither of these studies specifically focused on aggression, making the present study the first to examine the sources of covariation between activity and childhood aggression.

### Affect-extraversion

Phenotypic correlations between affect-extraversion and aggression negatively increased from 6 to 36 months. Affect-extraversion began to significantly correlate with aggression at 30 months, suggesting that a child’s sociability starts to relate to their tendency towards aggression at the end of toddlerhood. These findings are consistent with longitudinal literature showing that infants, toddlers, and preschoolers who are more socially withdrawn exhibit more externalizing behavior problems in early childhood (Chen et al., 2015; Guedeney et al., 2014; Liang et al., 2019). In line with these studies, we found that both baseline level and increases in a child’s affect-extraversion from 6 to 36 months were negatively associated with childhood aggression. These results, however, are inconsistent with one study that found no significant differences in externalizing behavior at age 7 among children with low, moderate, or high sociability trajectories from ages 2 to 4 years (Kim, 2022). This discrepancy may be attributed to the different time frames at which sociability was measured, as the present study used affect-extraversion scores from 6 to 36 months instead of 24 to 48 months. That is, negative associations between sociability trajectories and aggression may be confined to late infancy and toddlerhood (i.e., 12 to 36 months), a period of substantial social and emotional development when children first begin to exhibit prosocial tendencies and extend their relationships to other people (Brownell, 2013; Paulus, 2018; Warneken & Tomasello, 2007). Furthermore, it may also be the case that our findings are specific to aggression rather than the broader externalizing outcomes examined in this study (Kim, 2022).

Genetic correlations between affect-extraversion and aggression increased over time, implying that genetic influences underlying sociability were increasingly associated with lower aggression. This increase was modest and could be due to unmodeled gene-environment correlation, that is, the nonrandom exposure to environments based on genetic inheritance. Infants and toddlers who are more sociable at younger ages may be more sociable at later ages because parents and caregivers may recognize and cultivate extraverted behaviors. In line with this interpretation is the finding that 40% of the phenotypic correlation between the slopes of affect-extraversion and aggression was attributed to nonshared environmental influences. For genetic behaviors that are reinforced by subsequent environmental exposures, nonshared environmental variance is expected to increase and become more stable over time (Beam & Turkheimer, 2013). Together, these results represent the first examination of the etiology of the association between sociability (i.e., affect-extraversion) and aggression. Future genetically informed studies will be needed to corroborate these findings.

### Task orientation

Phenotypic correlations between task orientation and aggression negatively increased from 6 to 36 months. Task orientation became significantly associated with aggression at 24 months, a correlation that emerged later in development than that of activity and earlier than that of affect-extraversion. These findings are consistent with longitudinal studies that have found significant negative associations between effortful control, measured during infancy and toddlerhood, and childhood aggression (Gartstein et al., 2012; Kochanska & Knaack, 2003; Murray & Kochanska, 2002). Importantly, the negative associations reported in the present study are also consistent with the only study to date that has used the Infant Behavior Record to examine the association between temperament (task persistence) and externalizing problems (Deater-Deckard et al., 2007). Moreover, we also found that variance in both baseline level and increases in task orientation, denoted by the intercept and slope of its developmental trajectory, respectively, were negatively associated with aggression. These findings are supported by literature showing that increases in self-control during infancy (Rhee et al., 2018) and increases in effortful control during early childhood (Lengua, 2006) are negatively associated with behavior problems.

Genetic correlations between task orientation and aggression increased from 6 to 36 months, suggesting that genetic influences related to task orientation may progressively be associated with lower aggression. The intercepts and slopes of task orientation also showed significant negative genetic correlations with aggression. These findings imply that children with a greater genetic predisposition for staying on task and subsequently improving in this ability are less likely to exhibit aggressive behavior in childhood. Similar to activity and affect-extraversion, nonshared environmental influences appeared to contribute to the phenotypic correlation between task orientation and aggression towards the end of toddlerhood (i.e., 36 months). This effect, however, was small and nonsignificant, with 83% phenotypic correlation still being explained by genetic influences. Together, these findings are consistent with literature showing a substantial genetic basis to the covariation between effortful control, including related traits (e.g., task persistence, inhibitory control), and aggression (Deater-Deckard et al., 2007; Gagne et al., 2011, 2020; Lemery-Chalfant et al., 2008). It should be noted, however, that our finding regarding the association between the slopes of task orientation and aggression is inconsistent with prior research that has found both genetic *and* shared environmental variance accounting for the association between increases in infant self-control and externalizing behavior (Rhee et al., 2018). This discrepancy may be attributed to the task orientation construct used in the present study, which included aspects of self-regulation, such as attention span (Matheny, 1980), that were not directly captured by the self-control measure utilized in the prior study (Rhee et al., 2018). The present study remains the first study to examine the etiology of the association between developmental increases in temperamental task orientation and childhood aggression.

### Developmental trends across relationships between temperament and aggression

One of our most consistent findings was that genetic influences almost fully explained the observed associations between each temperament dimension and aggression. Notably, among these genetic components, most of the variance was unique, with peak genetic correlations ranging in magnitudes from .33 to .51. Thus, although temperament and aggression appear to have largely independent genetic bases, the genetic factors that they do have in common almost entirely account for their phenotypic correlations. As such, the progressive overlap in the genetic influences underlying each temperament dimension and aggression probably drives their increasing phenotypic correlations. An alternative explanation is one we posited above, in which gene-environment correlation may explain the increase in the genetic correlations between each temperament dimension and childhood aggression.

### Sex differences in relationships between temperament and aggression

The associations between each temperament dimension and aggression exhibited quantitative sex differences, in which boys and girls differed significantly in the magnitudes of genetic and environmental influences underlying each of these associations. Nonshared environmental influences appeared to play a greater role in the association between each temperament dimension and aggression in girls than boys. This trend began to occur late in toddlerhood (i.e., ages 30 and 36 months), which coincides with when children become more socially aware, learn cooperation and sharing skills, and start forming more complex relationships with peers (Malik & Marwaha, 2022). Our findings suggest that environmental factors (e.g., formation of sophisticated peer relations, differential parental treatment) influencing girls’ temperamental activity, sociability, and self-regulation may also influence their risk for aggression. Moreover, it appears that environmentally influenced increases in affect-extraversion (i.e., sociability) are a unique correlate of aggression in girls but not boys. A possible explanation for this finding may be that aggressive girls tend to exhibit greater levels of indirect and relational forms of aggression than aggressive boys (Björkqvist, 2018; Chamberlain, 2003; Taylor & Borduin, 2014). Consistent with this notion, of the children who endorsed ≥5 aggression items in our sample, girls had significantly higher mean scores than boys (*t*(57) = 2.60, *p* = .01) on items pertaining to indirect and/or relational aggression (“is bossy with other children,” “tries to get other children into trouble,” “gives other children dirty looks,” and “does not take orders when other children are in charge”). Future research should aim to replicate our findings in larger sample sizes and across older age cohorts to further elucidate the role of sex in the association between early-life temperament and childhood aggression.

### Limitations

The present study was limited by its modest sample size, which may have resulted in inadequate power to detect early-emerging associations between temperament and childhood aggression, particularly within the first year of life, that had been previously reported in the literature (Crockenberg et al., 2008; Lahey et al., 2008; Morales et al., 2022). Likewise, this study may have lacked sufficient power to detect shared environmental influences underlying the associations between task orientation (i.e., self-regulation) and aggression, as reported in a prior longitudinal study examining self-control and externalizing behavior (Rhee et al., 2018). Moreover, although the temperament and aggression scales used in the present study were shown to be reliable in our sample, both the Infant Behavior Record and the School Behavior Checklist are no longer in use today and have since been replaced by contemporary scales (e.g., the Infant Behavior Questionnaire and the Reactive and Proactive Aggression Questionnaire) that capture different dimensions of temperament (e.g., negative emotionality and surgency) and subtypes of aggression (e.g., proactive vs. reactive aggression). Thus, our ability to consider these aspects of temperament and aggression was limited. Lastly, this study had a predominantly White (80%) sample, which serves as a barrier to the generalizability of our findings to other racial and ethnic populations.

### Future directions

We encourage future studies to further elucidate the association between early-life temperament and aggression by examining trajectories of aggressive behavior in childhood. It has been shown that children at highest risk for externalizing problems and disorders in adulthood are those who exhibit rising trajectories of high physical aggression in early childhood rather than normative decreases observed during this time period (Reef et al., 2011; Tremblay et al., 2004). Given the longitudinal associations between early-life temperament and childhood aggression reported in the present study, future studies should examine whether level and change of infant and toddler temperament correlate with trajectories of aggression. Stratifying these analyses by physical and relational aggression will be especially important, as relational aggression exhibits a different developmental trajectory, emerging at age 30 months and increasing into middle childhood and adolescence (Archer & Coyne, 2005; Crick et al., 2006). Lastly, we encourage future studies to adopt genetically informative designs, which will be central to determining if varying etiological influences underlie associations between temperament and different subtypes of aggression.

## Conclusion

The present study examined the ages at which three dimensions of infant and toddler temperament – activity, affect-extraversion, and task orientation – became significant correlates of childhood aggression, supporting existing literature showing that activity, social withdrawal, and effortful control in the first two years of life bear significant associations with childhood externalizing outcomes (Gartstein et al., 2012; Guedeney et al., 2014; Lahey et al., 2008). This was the first study to explore and show that differences in mean-level increases in activity, affect-extraversion, and task orientation from age 6 to 36 months were significantly associated with childhood aggression. We found that common genetic influences primarily accounted for the associations between temperament and aggression throughout development, with nonshared environmental influences beginning to explain part of each association by 36 months. Finally, sex difference analyses revealed that nonshared environmental influences appeared to be more important for girls than boys, accounting for a greater proportion of each temperament-aggression association.

## Supporting information

Penichet et al. supplementary material Penichet et al. supplementary material

## Supplementary material

The supplementary material for this article can be found at https://doi.org/10.1017/S0954579424000634.
