# Supplementary material for: A genetically informed longitudinal study of early-life temperament and childhood aggression
Source: Dev Psychopathol. 2024 Apr 1;37(2):779–801. doi: 10.1017/S0954579424000634 (PMC12272078; doi:10.1017/S0954579424000634)
Supplement: Penichet et al. supplementary material [file S0954579424000634sup001.docx]

Supplementary Material: Appendix 1

**Table A1**

*Longitudinal Participation and Retention Rate at Age 7*

| Time Point | Sample Size | Participated at Age 7 |
| --- | --- | --- |
| 6 months | 448 | 252 (56.2%) |
| 12 months | 540 | 302 (55.9%) |
| 18 months | 512 | 299 (58.4%) |
| 24 months | 519 | 315 (60.7%) |
| 30 months | 488 | 309 (63.3%) |
| 36 months | 471 | 320 (67.9%) |

Supplementary Material: Appendix 2

**Table A2**

*Comparative analysis of participant attrition over time*

| Variable | Time Point | Participated at Age 7 | Dropped Out  (no data at Age 7) | Levene’s test | | *t* test | | Chi-squared test | |
| --- | --- | --- | --- | --- | --- | --- | --- | --- | --- |
| **Demographics** |  |  |  | *F* | *p* | *t* | *p* | *X^2^* | *p* |
| Gender (% Male) | 6 mo | 46% | 49% | - | - | - | - | 0.40 | 0.53 |
|  | 12 mo | 45% | 49% | - | - | - | - | 0.62 | 0.43 |
|  | 18 mo | 47% | 50% | - | - | - | - | 0.36 | 0.55 |
|  | 24 mo | 45% | 50% | - | - | - | - | 0.68 | 0.41 |
|  | 30 mo | 46% | 49% | - | - | - | - | 0.29 | 0.59 |
|  | 36 mo | 47% | 51% | - | - | - | - | 0.54 | 0.46 |
| SES | 6 mo | 53.07 (25.42) | 48.94 (28.29) | 8.40 | **0.00** | -1.60 | 0.11 | - | - |
|  | 12 mo | 54.14 (25.10) | 43.25 (28.43) | 6.50 | **0.01** | -0.38 | 0.71 | - | - |
|  | 18mo | 54.83 (24.66) | 51.32 (28.83) | 13.87 | **0.00** | -1.44 | 0.15 | - | - |
|  | 24 mo | 53.96 (24.88) | 51.01 (29.39) | 15.64 | **0.00** | -1.18 | 0.24 | - | - |
|  | 30 mo | 53.91 (24.89) | 53.27 (28.15) | 7.18 | **0.01** | -0.25 | 0.80 | - | - |
|  | 36 mo | 54.20 (24.77) | 52.68 (28.56) | 8.97 | **0.00** | -0.56 | 0.57 | - | - |
| **Temperament** |  |  |  |  |  |  |  |  |  |
| Activity | 6 mo | 0.23 (0.89) | 0.20 (0.90) | 0.08 | 0.78 | -0.31 | 0.76 | - | - |
|  | 12 mo | 0.57 (0.90) | 0.49 (0.87) | 0.41 | 0.52 | -1.01 | 0.31 | - | - |
|  | 18 mo | 0.86 (0.98) | 0.85 (1.01) | 0.10 | 0.76 | -0.04 | 0.96 | - | - |
|  | 24 mo | 0.78 (0.91) | 0.81 (0.94) | 0.86 | 0.35 | 0.32 | 0.75 | - | - |
|  | 30 mo | 0.87 (0.94) | 0.94 (0.97) | 1.28 | 0.26 | 0.71 | 0.48 | - | - |
|  | 36 mo | N/A | N/A | N/A | N/A | N/A | N/A | - | - |
| Affect-Extraversion | 6 mo | 0.11 (1.08) | 0.04 (1.09 | 0.02 | 0.89 | -0.65 | 0.52 | - | - |
|  | 12 mo | -0.09 (0.93) | -0.14 (0.99) | 1.01 | 0.32 | -0.58 | 0.56 | - | - |
|  | 18 mo | -0.34 (0.98) | -0.36 (0.96) | 0.43 | 0.51 | -0.22 | 0.83 | - | - |
|  | 24 mo | 0.25 (1.02) | 0.09 (0.99) | 0.25 | 0.62 | -1.77 | 0.08 | - | - |
|  | 30 mo | 0.29 (0.89) | 0.09 (0.87) | 0.00 | 0.97 | -2.42 | **0.02** | - | - |
|  | 36 mo | 0.36 (0.86) | 0.30 (0.97) | 3.53 | 0.06 | -0.66 | 0.51 | - | - |
| Task Orientation | 6 mo | 0.35 (0.92) | 0.23 (0.93) | 0.09 | 0.77 | -1.36 | 0.18 | - | - |
|  | 12 mo | 0.75 (0.73) | 0.57 (0.84) | 5.18 | **0.02** | -2.61 | **0.01** | - | - |
|  | 18 mo | 0.72 (0.85) | 0.57 (0.89) | 0.92 | 0.34 | -1.89 | 0.06 | - | - |
|  | 24 mo | 1.18 (0.88) | 0.92 (0.86) | 0.02 | 0.90 | -3.31 | **0.00** | - | - |
|  | 30 mo | 0.84 (0.90) | 0.70 (0.87) | 0.05 | 0.82 | -1.67 | 0.09 | - | - |
|  | 36 mo | 1.13 (0.91) | 1.02 (0.93) | 0.22 | 0.64 | -1.20 | 0.23 | - | - |

*Note.* Values in parentheses indicate standard deviations. **Bolded values** indicate statistical significance at an alpha level of less than 0.05. Welch's t-test was used when variances were significantly different.

Supplementary Material: Appendix 3

**Table A3**

*Bivariate Model Sample Sizes*

| Time Point | Sample Size |
| --- | --- |
| 6 months | N = 532  (251 male, 281 female)  116 MZ twin pairs  150 DZ twin pairs  (73 same-sex, 77 opposite sex) |
| 12 months | N = 572  (271 male, 301 female)  126 MZ twin pairs  160 DZ twin pairs  (77 same-sex, 73 opposite sex) |
| 18 months | N = 548  (261 male, 287 female)  124 MZ twin pairs  150 DZ twin pairs  (71 same-sex, 79 opposite sex) |
| 24 months | N = 538  (257 male, 281 female)  121 MZ twin pairs  148 DZ twin pairs  (67 same-sex, 81 opposite sex) |
| 30 months | N = 512  (241 male, 271 female)  118 MZ twin pairs  138 DZ twin pairs  (65 same-sex, 73 opposite sex) |
| 36 months | N = 484  (230 male, 254 female)  115 MZ twin pairs  127 DZ twin pairs  (61 same-sex, 66 opposite sex) |

Supplementary Material: Appendix 4

**Table A4**

*Final MNFLA model results and covariate effects*

|  |  | Covariate effects | | |
| --- | --- | --- | --- | --- |
| Reference Parameter | Baseline | Age | Sex | Age x Sex |
| Activity |  |  |  |  |
| *Mean* | 0.00^a^ | 0.02** (0.004) | - | 0.01*** (0.002) |
| *Variance* | 1.00^a^ | - | - | - |
| *IBR 14. Amount of Gross*  *Bodily Movement* | 8.50 (2.36) | - | - | - |
| *IBR 21. Body Motion* | 3.99 (0.29) | -0.02* (0.009) | - | -0.01* (0.005) |
| *IBR 25. Level of Energy* | 1.76 (0.10) | - | - | - |
| Affect-Extraversion |  |  |  |  |
| Mean | 0.00^a^ | - | - | - |
| Variance | 1.00^a^ | - | - | - |
| *IBR 1. Responsiveness to*  *Persons* | 0.91 (0.10) | - | - | - |
| *IBR 2. Responsiveness to*  *Examiner* | 1.35 (0.12) | -0.03*** (0.008) | - | - |
| *IBR 4. Cooperativeness* | 2.20 (0.17) | -0.03** (0.010) |  | - |
| *IBR 5. Fearfulness* | -0.69 (0.10) | 0.05*** (0.008) | - | - |
| *IBR 7. Degree of*  *Happiness* | 3.64 (0.35) | - | - | - |
| *IBR 10. Object*  *rientation* | -0.207 (0.10) | - | - | - |
| *IBR 13. Endurance* | 2.28 (0.17) |  | - | - |
| Task Orientation |  |  |  |  |
| Mean | 0.00^a^ | 0.04*** (0.004) | - | - |
| Variance | 1.00^a^ | - | - | - |
| *IBR 8. Responsiveness to*  *Objects; toys, test materials* | 2.60 (0.20) | 0.04*** (0.012) | - | - |
| *IBR 11. Goal Directedness* | 3.94 (0.38) | - | - | - |
| *IBR 21. Attention Span* | 2.93 (0.22) | - | - | - |

*Note.* IBR = Infant Behavior Record; **^†^** 0.05 < p < 0.10, * p < 0.05, ** p < 0.01, *** p < 0.001

Supplementary Material: Appendix 5

**Table A5**

*Model fit statistics for three growth curve models of the three temperament dimensions*

| Temperament Dimension | Model | df | χ2 | CFI | TLI | RMSEA |
| --- | --- | --- | --- | --- | --- | --- |
| Activity | **FSL** | **16** | **30.37** | **0.92** | **0.87** | **0.039** |
|  | LG | 19 | 66.35 | 0.73 | 0.64 | 0.064 |
|  | QG | 17 | 40.36 | 0.86 | 0.80 | 0.048 |
| Affect-Extraversion | **FSL** | **24** | **110.12** | **0.61** | **0.47** | **0.077** |
|  | LG | 28 | 180.05 | 0.67 | 0.17 | 0.096 |
|  | QG | 26 | 155.61 | 0.42 | 0.26 | 0.087 |
| Task Orientation | **FSL** | **24** | **66.00** | **0.89** | **0.85** | **0.055** |
|  | LG | 28 | 152.45 | 0.68 | 0.62 | 0.078 |
|  | QG | 26 | 131.45 | 0.73 | 0.65 | 0.082 |

*Note.* The model in bold signifies the best fitting model. FSL = ‘flexible slope’ latent growth curve model; LG = linear growth curve model; QG = quadratic growth curve model. df = degrees of freedom; χ2 = chi-square; CFI = Comparative Fit Index; TLI = Tucker Lewis Index; RMSEA = Root Mean Square Error of Approximation

Supplementary Material: Appendix 6

**Table A6**

*Phenotypic correlations between temperament from 6 to 36 months and variables from the School Behavior Checklist at age 7*

|  | Aggression | | |
| --- | --- | --- | --- |
|  | Activity | Affect-Extraversion | Task Orientation |
| 6 months | -.01 [-.11, .09] | -.05 [-.15, .05] | -.05 [-.16, .05] |
| 12 months | .08 [-.02, .18] | .02 [-.09, .13] | .02 [-.09, .13] |
| 18 months | **.18** [.08, .28] | -.04 [-.15, .06] | -.07 [-.18, .04] |
| 24 months | **.18** [.09, .28] | -.10 [-.20, -.00] | -.12 [-.21, -.02] |
| 30 months | **.26** [.16, .35] | **-.20** [-.30, -.10] | **-.20** [-.30, -.11] |
| 36 months | N/A | -.12 [-.23, -.01] | **-.23** [-.33, -.13] |
| Intercept | .05 [-.05, .15] | **-.16** [-.26, -.07] | **-.21** [-.30, -.12] |
| Slope | **.23** [.14, .32] | **-.15** [-.24, -.06] | **-.17** [-.27, -.08] |
|  | Low Need Achievement | | |
|  | Activity | Affect-Extraversion | Task Orientation |
| 6 months | -.02 [-.13, .09] | **-.17** [-.27, -.07] | **-.16** [-.27, -.05] |
| 12 months | -.03 [-.13, .07] | -.01 [-.11, .10] | -.08 [-.19, .03] |
| 18 months | .07 [-.02, .17] | **-.16** [-.26, -.07] | **-.21** [-.31, -.11] |
| 24 months | .06 [-.04, .16] | **-.16** [-.25, -.06] | **-.25** [-.34, -.16] |
| 30 months | .13 [.03, .23] | **-.19** [-.28, -.09] | **-.23** [-.32, -.13] |
| 36 months | N/A | **-.17** [-.27, -.06] | **-.23** [-.32, -.13] |
| Intercept | -.01 [-.11, .08] | **-.25** [-.34, -.16] | **-.32** [-.40, -.23] |
| Slope | .13 [.04, .22] | -.12 [-.21, -.03] | **-.31** [-.40, -.22] |
|  | Anxiety | | |
|  | Activity | Affect-Extraversion | Task Orientation |
| 6 months | .07 [-.04, .18] | -.00 [-.11, .11] | .02 [-.10, .13] |
| 12 months | .06 [-.04, .16] | -.04 [-.15, .06] | -.09 [-.19, .02] |
| 18 months | .05 [-.05, .15] | **-.16** [-.26, -.06] | -.13 [-.24, -.03] |
| 24 months | -.05 [-.15, .05] | **-.14** [-.23, -.04] | **-.15** [-.24, -.06] |
| 30 months | -.02 [-.12, .08] | -.13 [-.23, -.04] | **-.14** [-.24, -.05] |
| 36 months | N/A | -.07 [-.17, .04] | -.09 [-.19, .01] |
| Intercept | .07 [-.02, .17] | **-.16** [-.26, -.07] | **-.17** [-.26, -.08] |
| Slope | -.03 [-.12, .06] | -.03 [-.12, .06] | **-.16** [-.25, -.07] |
|  | Academic Disability | | |
|  | Activity | Affect-Extraversion | Task Orientation |
| 6 months | -.05 [-.16, .06] | .01 [-.10, .12] | -.05 [-.16, .06] |
| 12 months | .01 [-.08, .11] | .04 [-.07, .14] | -.01 [-.12, .10] |
| 18 months | .07 [-.02, .17] | -.11 [-.20, -.01] | -.03 [-.13, .08] |
| 24 months | .08 [-.02, .17] | -.03 [-.12, .06] | .01 [-.08, .11] |
| 30 months | **.14** [.05, .24] | -.08 [-.17, .02] | -.13 [-.23, -.04] |
| 36 months | N/A | -.12 [-.22, -.02] | **-.17** [-.27, -.08] |
| Intercept | -.01 [-.11, .08] | -.11 [-.20, -.01] | -.11 [-.20, -.02] |
| Slope | **.14** [.05, .23] | -.07 [-.16, .02] | -.10 [-.19, -.01] |
|  | Extraversion | | |
|  | Activity | Affect-Extraversion | Task Orientation |
| 6 months | .07 [-.03, .17] | .07 [-.03, .17] | .04 [-.06, .14] |
| 12 months | .01 [-.09, .11] | .01 [-.10, .11] | -.05 [-.17, .06] |
| 18 months | -.06 [-.17, .04] | .03 [-.08, .13] | .07 [-.04, .18] |
| 24 months | -.07 [-.17, .03] | .03 [-.07, .12] | .11 [.01, .20] |
| 30 months | -.10 [-.21, -.00] | .10 [.00, .21] | **.15** [.05, .24] |
| 36 months | N/A | .08 [-.02, .19] | **.15** [.05, .25] |
| Intercept | .05 [-.05, .15] | .09 [-.01, .19] | **.15** [.05, .24] |
| Slope | -.13 [-.22, -.04] | .08 [-.02, .17] | .12 [.03, .22] |

*Note.* Values in brackets represent 95% confidence intervals. **Bolded values** indicate statistical significance at an alpha level of less than 0.05. False Discovery Rate (FDR) correction was employed to adjust for multiple testing.

Supplementary Material: Appendix 7

**Table A7**

*Phenotypic correlations between temperament dimensions at each time point*

|  | 6 months | 12 months | 18 months | 24 months | 30 months | 36 months |
| --- | --- | --- | --- | --- | --- | --- |
| Activity*–*  Affect-Extraversion | .06  [-.02, .14] | **-.13**  [-.20, -.05] | **-.24**  [-.31, -.17] | **-.31**  [-.38, -.24] | **-.22**  [-.30, -.15] | N/A |
| Activity–  Task Orientation | **.27**  [.19, .34] | -.03  [-.11, .04] | **-.20**  [-.27, -.12] | **-.30**  [-.37, -.24] | **-.38**  [-.45, -.32] | N/A |
| Affect-Extraversion–Task Orientation | **.56**  [.51, .61] | **.46**  [.40, .51] | **.52**  [.47, .58] | **.62**  [.58, .67] | **.71**  [.67, .75] | **.65**  [.60, .70] |

*Note.* Values in brackets represent 95% confidence intervals. **Bolded values** indicate statistical significance at an alpha level of less than 0.05.

Supplementary Material: Appendix 8

**Table A8**

*Standardized Path Coefficients from Bivariate ACE Models for Task Orientation and Aggression*

|  | Estimate | S.E. | Est./S.E. | P-Value |
| --- | --- | --- | --- | --- |
| **6 months** |  |  |  |  |
| A1 → Task Orientation | -0.17 | 0.16 | -1.04 | 0.30 |
| A1 → Aggression | -0.75 | 0.08 | -9.82 | 0.00 |
| C1 → Task Orientation | -0.58 | 0.06 | -10.01 | 0.00 |
| C1 → Aggression | 0.30 | 0.16 | 1.84 | 0.07 |
| E1 → Task Orientation | 0.79 | 0.04 | 20.57 | 0.00 |
| E1 → Aggression | 0.00 | 0.06 | -0.03 | 0.97 |
| A2 → Aggression | 0.00 | 3.95 | 0.00 | 1.00 |
| C2 → Aggression | 0.00 | 0.55 | 0.00 | 1.00 |
| E2 → Aggression | 0.53 | 0.05 | 10.97 | 0.00 |
| **12 months** |  |  |  |  |
| A1 → Task Orientation | 0.58 | 0.11 | 5.26 | 0.00 |
| A1 → Aggression | -0.02 | 0.19 | -0.10 | 0.92 |
| C1 → Task Orientation | 0.14 | 0.34 | 0.43 | 0.67 |
| C1 → Aggression | -0.23 | 0.31 | -0.75 | 0.45 |
| E1 → Task Orientation | 0.72 | 0.05 | 16.06 | 0.00 |
| E1 → Aggression | 0.09 | 0.05 | 1.66 | 0.10 |
| A2 → Aggression | 0.78 | 0.10 | 7.72 | 0.00 |
| C2 → Aggression | 0.00 | 1.16 | 0.00 | 1.00 |
| E2 → Aggression | 0.51 | 0.05 | 10.90 | 0.00 |
| **18 months** |  |  |  |  |
| A1 → Task Orientation | 0.47 | 0.22 | 2.18 | 0.03 |
| A1 → Aggression | -0.28 | 0.36 | -0.77 | 0.44 |
| C1 → Task Orientation | 0.34 | 0.23 | 1.48 | 0.14 |
| C1 → Aggression | 0.11 | 0.40 | 0.27 | 0.78 |
| E1 → Task Orientation | 0.75 | 0.05 | 14.56 | 0.00 |
| E1 → Aggression | 0.04 | 0.06 | 0.73 | 0.47 |
| A2 → Aggression | -0.74 | 0.18 | -4.12 | 0.00 |
| C2 → Aggression | -0.16 | 0.57 | -0.28 | 0.78 |
| E2 → Aggression | 0.52 | 0.05 | 10.92 | 0.00 |
| **24 months** |  |  |  |  |
| A1 → Task Orientation | 0.39 | 0.26 | 1.51 | 0.13 |
| A1 → Aggression | -0.47 | 0.48 | -0.98 | 0.33 |
| C1 → Task Orientation | 0.39 | 0.20 | 1.93 | 0.05 |
| C1 → Aggression | 0.13 | 0.33 | 0.40 | 0.69 |
| E1 → Task Orientation | 0.78 | 0.05 | 15.90 | 0.00 |
| E1 → Aggression | 0.04 | 0.06 | 0.60 | 0.55 |
| A2 → Aggression | -0.63 | 0.38 | -1.64 | 0.10 |
| C2 → Aggression | 0.15 | 0.62 | 0.25 | 0.81 |
| E2 → Aggression | 0.52 | 0.05 | 10.91 | 0.00 |
| **30 months** |  |  |  |  |
| A1 → Task Orientation | 0.52 | 0.12 | 4.17 | 0.00 |
| A1 → Aggression | -0.47 | 0.28 | -1.66 | 0.10 |
| C1 → Task Orientation | 0.16 | 0.27 | 0.62 | 0.54 |
| C1 → Aggression | 0.28 | 0.24 | 1.17 | 0.24 |
| E1 → Task Orientation | 0.82 | 0.05 | 17.81 | 0.00 |
| E1 → Aggression | 0.01 | 0.06 | 0.18 | 0.86 |
| A2 → Aggression | -0.60 | 0.28 | -2.16 | 0.03 |
| C2 → Aggression | 0.00 | 0.70 | 0.00 | 1.00 |
| E2 → Aggression | 0.52 | 0.05 | 10.87 | 0.00 |
| **36 months** |  |  |  |  |
| A1 → Task Orientation | 0.73 | 0.07 | 9.83 | 0.00 |
| A1 → Aggression | -0.32 | 0.15 | -2.10 | 0.04 |
| C1 → Task Orientation | -0.19 | 0.24 | -0.81 | 0.42 |
| C1 → Aggression | -0.29 | 0.22 | -1.32 | 0.19 |
| E1 → Task Orientation | 0.60 | 0.04 | 14.59 | 0.00 |
| E1 → Aggression | -0.06 | 0.06 | -1.04 | 0.30 |
| A2 → Aggression | 0.70 | 0.14 | 5.14 | 0.00 |
| C2 → Aggression | 0.00 | 0.65 | 0.00 | 1.00 |
| E2 → Aggression | 0.52 | 0.05 | 10.98 | 0.00 |
| **Intercept** |  |  |  |  |
| A1 → Task Orientation | -0.50 | 0.07 | -7.48 | 0.00 |
| A1 → Aggression | 0.42 | 0.15 | 2.88 | 0.00 |
| C1 → Task Orientation | -0.24 | 0.11 | -2.12 | 0.03 |
| C1 → Aggression | -0.24 | 0.21 | -1.15 | 0.25 |
| E1 → Task Orientation | -0.39 | 0.03 | -13.26 | 0.00 |
| E1 → Aggression | -0.05 | 0.06 | -0.80 | 0.42 |
| A2 → Aggression | -0.66 | 0.16 | -4.21 | 0.00 |
| C2 → Aggression | 0.00 | 0.77 | 0.00 | 1.00 |
| E2 → Aggression | 0.52 | 0.05 | 11.03 | 0.00 |
| **Slope** |  |  |  |  |
| A1 → Task Orientation | -0.51 | 0.09 | -5.66 | 0.00 |
| A1 → Aggression | 0.42 | 0.22 | 1.95 | 0.05 |
| C1 → Task Orientation | -0.35 | 0.11 | -3.09 | 0.00 |
| C1 → Aggression | -0.15 | 0.28 | -0.54 | 0.59 |
| E1 → Task Orientation | -0.47 | 0.03 | -13.74 | 0.00 |
| E1 → Aggression | -0.07 | 0.06 | -1.20 | 0.23 |
| A2 → Aggression | 0.68 | 0.17 | 3.94 | 0.00 |
| C2 → Aggression | -0.03 | 3.10 | -0.01 | 0.99 |
| E2 → Aggression | -0.51 | 0.05 | -10.95 | 0.00 |

*Note.* Latent variables (A1, C1, E1, A2, C2, E2) are denoted on Figure 1.
